# Tautomerism and antioxidant activity of some 4-acylpyrazolone-based Schiff bases: a theoretical study†

**DOI:** 10.1039/c8ra05987j

**Published:** 2018-09-03

**Authors:** Esam A. Orabi

**Affiliations:** Department of Chemistry, Faculty of Science, Assiut University Assiut 71516 Egypt e_orabi@live.concordia.ca orabiesam@gmail.com +20-514-848-2868 +20-514-848-2424 extn 5835

## Abstract

4-Acylpyrazolone Schiff bases display antimicrobial, antiprion, antioxidant, and other biological activities. They are also used as ligands and some of their complexes possess photoluminescence and anticancer properties. These Schiff bases may exist in four tautomeric forms that correspond to H at the C (imine-one(I)), N (imine-one(II)), and O (imine-ol) atoms of the pyrazolone ring or at the azomethine N atom (amine-one). While crystal structures show the amine-one form, the identity of the tautomeric form in solution and the structure–antioxidant activity relationship of these compounds are not clear. We perform quantum mechanical investigations on nine 4-acylpyrazolone-based Schiff bases at the B3LYP/6-311++G(d,p) level of theory in the gas phase and in chloroform, dimethyl sulfoxide, and water using the polarizable continuum model (PCM). Results show that the imine-ol, imine-one(I), and imine-one(II) isomers are, in respective, 6.5–8.0, 17–20, and 19–23 kcal mol^−1^ less stable than the amine-one form and that solvents further stabilize the later form. The energy barrier for imine-ol to amine-one conversion is only 0–1 kcal mol^−1^, showing that formation of the latter form is both kinetically and thermodynamically favorable. NMR calculations show that H in the amine-one and imine-ol forms appears at *δ* = 11.9–12.9 and 14.0–15.7 ppm, respectively, revealing that the experimentally reported ^1^H NMR spectra of these compounds are due to the amine-one tautomeric form. The structure–antioxidant activity relationship is investigated and structural modifications that increase the antioxidant activity are discussed. Calculations using the PCM show that the vertical ionization potential (IPV) is inversely proportional with the ferric reducing antioxidant power (FRAP) of these compounds. IPV thus presents a valuable tool for predicting the FRAP.

## Abstract 2

Computational investigations reveal that 4-acylpyrazolone Schiff bases adopt the amine-one structure and that ionization potentials provide a measure for their antioxidant activity.

## Graphical abstract

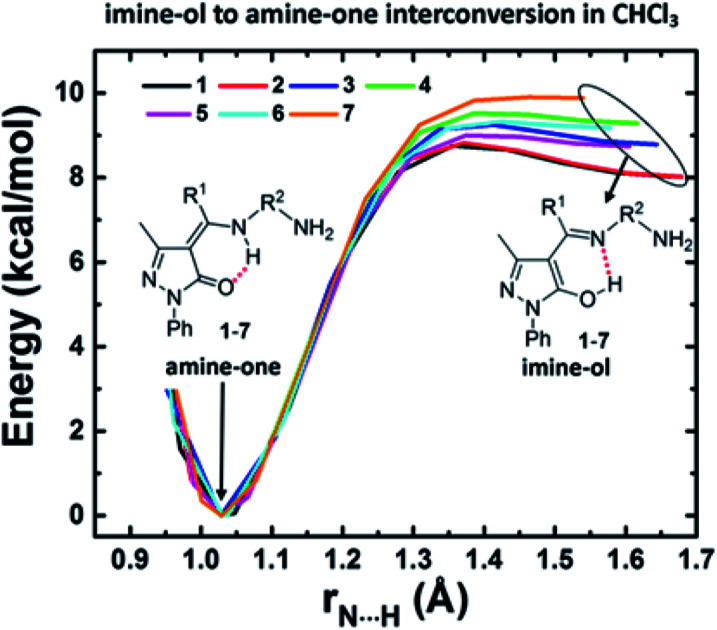

## Introduction

The first pyrazolone compound was synthesized by Knorr in 1883 through a condensation reaction between ethyl acetoacetate and phenylhydrazine.^1^ The *N*-methyl derivative of this compound was then prepared and found to possess analgesic and antipyretic effects.^2^ Other derivatives that possess analgesic,^3^ antimicrobial,^4^ anti-inflammatory,^5^ antioxidant,^6^ and antiprion^7^ activities have also been reported. Some pyrazolones have been used as fungicides and herbicides for crop protection, as dyes for cotton, wool, and silk,^3^ as ligands for synthesis of metal complexes,^8^ and as corrosion inhibitors.^9^

**Fig. 1:**
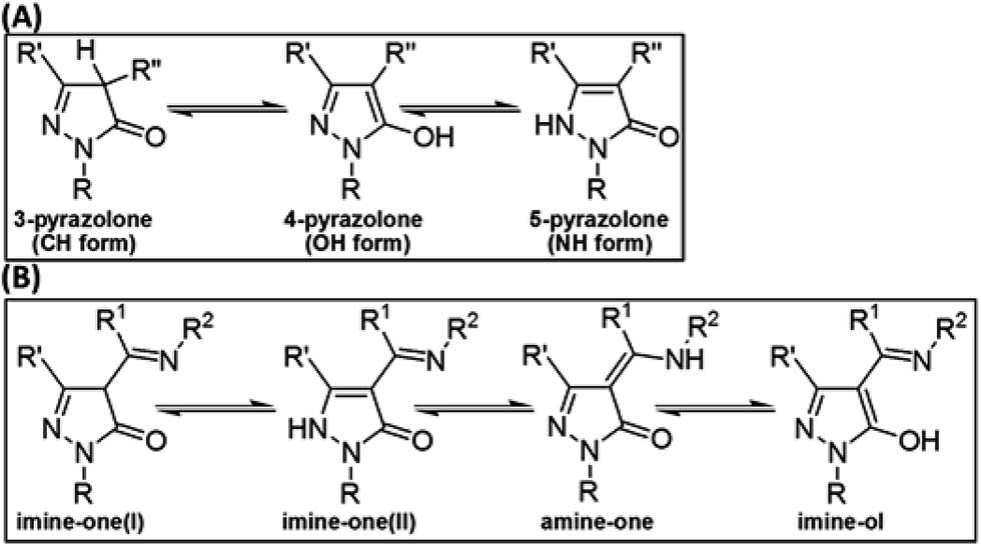
Tautomerism in pyrazolones (A) and in 4-acylpyrazolone Schiff bases (B). R, R′, R′′, R^1^, and R^2^ are H atoms or general substituents.

Pyrazolones can exist in three tautomeric forms (Fig. 1A) known as 3-pyrazolone (CH form), 4-pyrazolone (OH form), and 5-pyrazolone (NH form).^6^ It was recently shown that the relative stability of these forms depends on the solvent and on substituents R′ and R′′, and that the presence of bulky or aromatic R′′ groups limits the formation of the CH isomer.^6^

Pyrazolones in which R′′ is an acyl group (R^1^CO) were found to possess multidrug resistance-modulating activity^10^ and the benzoyl derivative (R^1^ = Ph) was shown to be a potential antiprion agent.^7^ 4-Acylpyrazolones represent an important class of chelating ligands known as heterocyclic β-diketones which have been employed for preparation of mono and dinuclear complexes.^11–14^ Among various biological activities, some of these complexes were shown to possess moderate to high anticancer activity.^13,14^

Schiff bases derived from the condensation of 4-acylpyrazolones with aliphatic or aromatic amines (R^2^NH_2_) have become the focus of many recent investigations.^15–21^ These studies focused on investigating the structure of these Schiff bases^15–20^ and in using them as ligands for the synthesis of various transition metal complexes.^15,16,19^ In addition to the three tautomeric forms characteristics for the pyrazolone ring (Fig. 1A), these Schiff bases stabilize a fourth tautomer with an amine (–NHR^2^) rather than imine (=NR^2^) group. We label the four tautomeric structures as imine-one(I), imine-one(II), imine-ol, and amine-one (Fig. 1B).

Although experiments are showing the amine-one structure in the solid state,^15–18,20^ contradictory results are reported in solution. ^1^H NMR spectra of 4-acylpyrazolone Schiff bases in chloroform and dimethyl sulfoxide (DMSO) indicate that they exist in the amine-one^15,17,19^ or imine-ol^16,21^ forms. Pyrazolone Schiff bases with no H on atoms immediately neighbouring to the NH and OH groups display a singlet peak for the latter polar H atoms and distinguishing the amine-one and imine-ol forms based on ^1^H NMR measurements is thought to be not possible.^15^ Computational investigations present an important alternative to experiments for determining the relative stability of the four isomeric forms and to study the impact of environment (solvent polarity) on their relative stability.

**Fig. 2:**
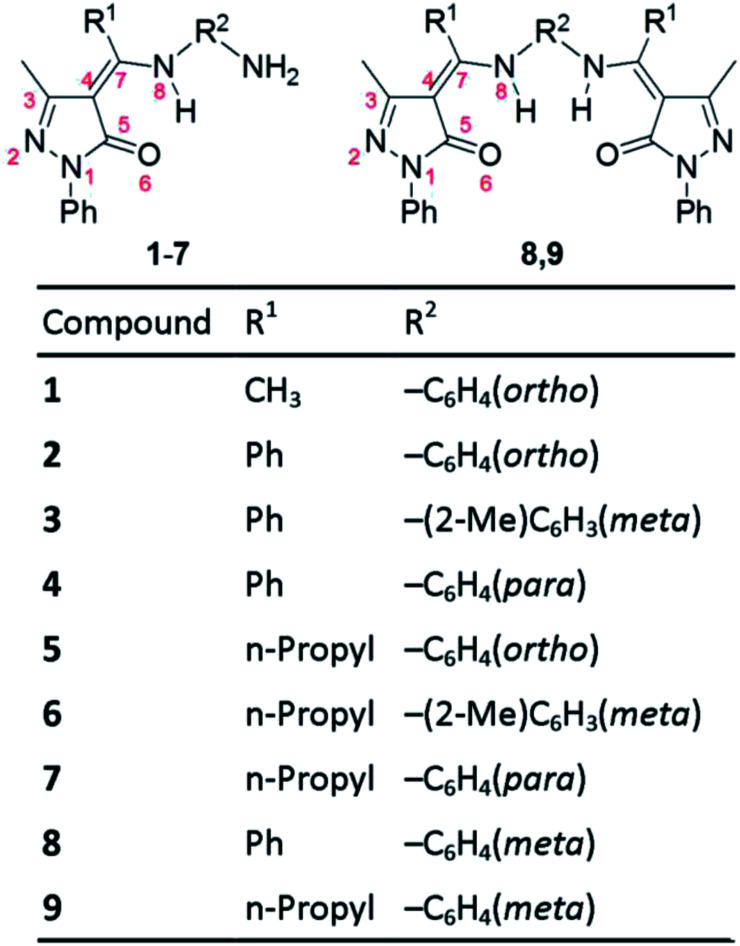
Structures of the nine pyrazolone Schiff bases considered in this work (presented in the amine-one tautomeric form). Certain atoms are numbered (in red) to facilitate discussing the structural properties of the four tautomeric forms. For synthesis and nomenclature of these compounds see ref. 21.

Recently, Parmar *et al.* synthesized nine 4-acylpyrazolone Schiff bases (Fig. 2) and suggested that they persist in the imine-ol tautomeric form in solution.^21^ They also showed that the compounds possess antibacterial, antifungal, and antioxidant activities.^21^ Interestingly, Schiff base 1 and 4 display the lowest and highest antioxidant activity and compounds 2 and 4, which differ only in the position of the NH_2_ group being *ortho* in 2 and *para* in 4, have different antioxidant activity.^21^ This indicates that not only the nature of substituents but also their location may significantly impact the biological behaviour of these Schiff bases. Understanding the structure–antioxidant function of these compounds is important for the design of more efficient antioxidants.

The aim of this work is to computationally investigate the relative stability of the four tautomeric forms in 4-acylpyrazolone-based Schiff bases in gas phase and in solvents of different polarity and to study the structure–antioxidant activity relationship of this family of compounds. For this regard, we use the nine pyrazolone Schiff bases synthesized by Parmar *et al.*^21^ (Fig. 2) as model compounds.

## Computational details

All quantum mechanical calculations are performed with the Gaussian 09 program^22^ using the density functional theory method with the B3LYP functional^23^ and the 6-311++G(d,p) basis set. The geometry of various plausible conformers of each of the four tautomeric forms of compounds 1–9 is optimized in the gas phase and only the most stable structure of each tautomer is reported. While the presence of two pyrazolone fragments in compounds 8 and 9 results in four tautomeric structures in each fragment and hence in sixteen possible isomers in total, only the four isomers with identical tautomeric forms in both fragments are considered.

Results show that imine-one(I, II) tautomers of all compounds are energetically unfavorable. Solvent effects are thus investigated by re-optimizing the gas phase structures of amine-one and imine-ol tautomers only, using the polarizable continuum model (PCM) with chloroform (*ε* = 4.7), DMSO (*ε* = 46.8), and water (*ε* = 78.4) as implicit solvents. Chloroform and DMSO are commonly used solvents in ^1^H NMR measurements^15–17,19,21^ and water is considered to extend examining the polarity-dependence of the calculated properties.

**Fig. 3:**
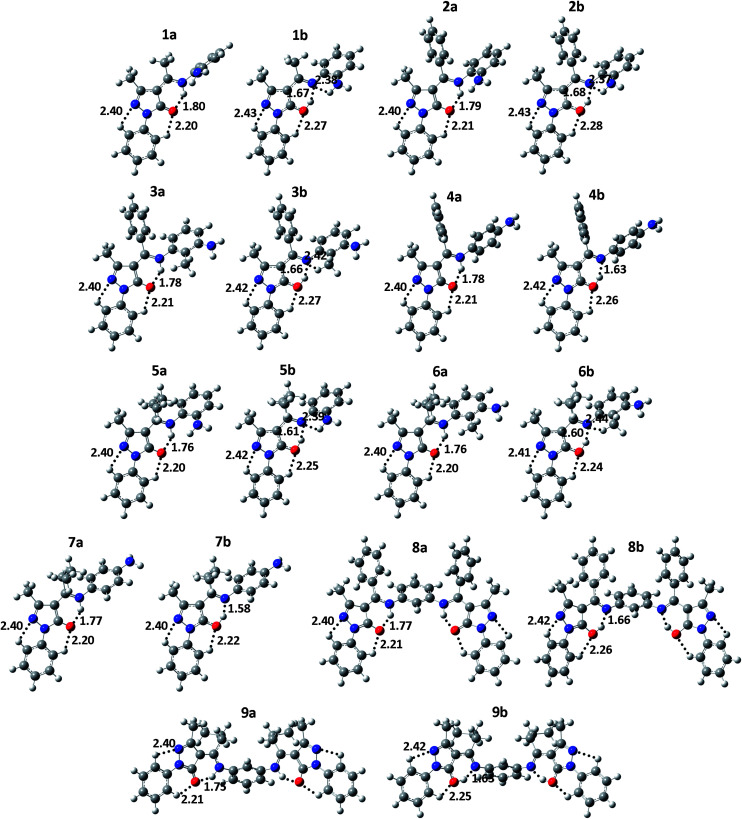
Optimized gas-phase geometry of the global minimum conformers of the amine-one (a) and imine-ol (b) tautomeric forms of 4-acylpyrazolone Schiff bases 1–9 at the B3LYP/6-311++G(d,p) level of theory. Atom color code: H (white), C (gray), N (blue), and O (red). Dotted lines designate intramolecular σ-type H-bonds and numbers indicate H-bond distances. Note that right and lift-side pyrazolone fragments of compounds 8 and 9 are characterized by the same H-bond distances and thus only those in the lift-side are presented. The atomic coordinates are provided in the ESI.†

Calculations reveal that the amine-one tautomeric form is more stable than the imine-ol form and that in both forms, N_8_ and O_6_ are directly H-bonded (see Fig. 3). To examine the energy barriers associated with interconversion of the two forms, relaxed potential energy scans are performed by decreasing the distance between N and H in the imine-ol form by 0.05 Å to a final distance of ∼0.90 Å. Due to the large size of compounds 8 and 9, the scan is performed only for compounds 1–7 in gas phase and in the three solvents. Optimization of the transition state (TS) structure along the amine-one to imine-ol interconversions of compounds 1 and 2 results in <0.05 kcal mol^−1^ difference in energy relative to the highest energy points along the relaxed scans. The latter points are thus considered as the TS and used to calculate the energy barriers for the interconversions in all compounds.

To determine the difference in chemical shifts for H bound to N_2_, C_4_, O_6_, and N_8_, we calculate the NMR chemical shifts of the four tautomeric forms of compounds 1–9 in the gas phase using the gauge invariant atomic orbitals (GIAO) method. The calculated isotropic magnetic shielding is used to calculate the scaled chemical shift as: *δ* = (31.984 – isotropic magnetic shielding)/1.0405, where 31.984 and 1.0405 are linear regression parameters.^24^ No NMR calculations are performed in presence of the solvents as, to the best of our knowledge, no correction parameters are available for these solvents at the model chemistry employed. Note that the accuracy of ^1^H NMR calculations have been shown to not depend on the cost of the calculation.^24^

Antioxidants delay or prevent cellular damage by reacting with free radicals at a rate faster than for free radical–substrate interaction. Of their reaction mechanisms, antioxidants may transfer H or both an electron and a proton to the free radical.^6,25^ It was shown that X–H (X = C, N or O) bond dissociation energy (BDE) and vertical ionization potentials (IPV) can be used to compare the rate of the two mechanisms and to compare compounds' antioxidant activities.^6^ BDEs are calculated as:

1 BDE = *E*_radical_ + *E*_H_ − *E*_molecule_

where *E*_molecule_, *E*_H_, and *E*_radical_ are the energies of the optimized Schiff base and its C_4_–H (imine-one(I) form), N_2_–H (imine-one(II) form), N_8_–H (amine-one form), or O–H (imine-ol form) homolytic bond cleavage products. Note that cleavage of either of the four bonds results in the same final product and thus the energy of the global minimum radical is used to calculate BDEs of the four bonds in each compound. Frequency calculations are performed on molecular and radical species to ensure they are energy minima (no imaginary frequencies) and to correct electronic energies for zero-point vibrational energies.

IPVs are calculated using the non-equilibrium polarizable continuum model (NEPCM) as reported elsewhere.^6^ In particular they represent the energy difference between radical cations and corresponding neutral molecules with energies of the formers evaluated while accounting for the fast solvent polarization but not the much slower geometry relaxation of the molecule or the reorientation of solvent molecules.^6^

## Results and discussion

### Properties of compounds 1–9 in the gas phase

The optimized geometries of the global minimum conformers of the amine-one and imine-ol isomeric forms of compounds 1–9 are given in Fig. 3 and their relative stability, IPV, BDE, dipole moment, and selected bond distances are listed in Table 1. Geometries and properties of the imine-one(I, II) forms and the total energy and atomic coordinates of the four tautomeric forms are presented in the ESI.†

**Table 1:** Relative stability (Δ*E*), vertical ionization potential (IPV), bond dissociation energy (BDE), dipole moment, and selected bond distances (*r*) of the global minimum amine-one and imine-ol tautomeric forms of compounds 1–9^a^

Property	1		2		3		4		5		6		7		8		9	
	Amine-one	Imine-ol	Amine-one	Imine-ol	Amine-one	Imine-ol	Amine-one	Imine-ol	Amine-one	Imine-ol	Amine-one	Imine-ol	Amine-one	Imine-ol	Amine-one	Imine-ol	Amine-one	Imine-ol
Δ*E*^b^	0.0 (0.0)	6.6 (6.0)	0.0 (0.0)	6.5 (6.0)	0.0 (0.0)	7.2 (6.5)	0.0 (0.0)	7.5 (6.8)	0.0 (0.0)	7.2 (6.5)	0.0 (0.0)	7.5 (6.6)	0.0 (0.0)	8.0 (7.0)	0.0 (0.0)	13.2 (11.9)	0.0 (0.0)	12.6 (11.3)
IPV	163.4	164.2	161.7	162.8	158.0	159.1	156.1	156.7	162.4	163.0	160.3	165.0	158.6	160.2	154.5	161.1	156.8	165.4
BDE^b^	93.0 (83.8)	86.4 (77.9)	91.1 (82.0)	84.6 (76.0)	96.3 (87.1)	89.1 (80.6)	96.2 (86.9)	88.7 (80.1)	93.7 (84.7)	86.6 (78.2)	97.4 (88.1)	89.9 (81.5)	96.5 (87.5)	88.5 (80.5)	95.8 (87.1)	82.6 (75.8)	96.2 (87.1)	83.6 (75.8)
Dipole	5.88	1.38	5.84	1.69	6.50	2.85	7.72	4.11	5.39	1.73	6.48	2.92	7.63	3.81	11.22	5.33	6.99	3.94
*r*_N_1_N_2__	1.392	1.385	1.394	1.387	1.393	1.387	1.393	1.387	1.391	1.386	1.391	1.386	1.390	1.386	1.393	1.387	1.391	1.385
*r*_N_2_C_3__	1.304	1.318	1.305	1.318	1.305	1.318	1.305	1.318	1.305	1.318	1.305	1.318	1.305	1.318	1.304	1.317	1.304	1.317
*r*_C_3_C_4__	1.446	1.433	1.446	1.435	1.447	1.435	1.446	1.435	1.447	1.436	1.447	1.435	1.447	1.435	1.448	1.435	1.449	1.436
*r*_C_4_C_5__	1.460	1.403	1.460	1.404	1.461	1.405	1.460	1.405	1.460	1.407	1.460	1.407	1.461	1.407	1.464	1.406	1.464	1.407
*r*_C_4_C_7__	1.392	1.447	1.391	1.446	1.394	1.447	1.394	1.447	1.395	1.447	1.397	1.449	1.397	1.448	1.390	1.445	1.392	1.447
*r*_C_5_O_6__	1.244	1.321	1.245	1.322	1.245	1.320	1.245	1.319	1.247	1.318	1.246	1.317	1.245	1.315	1.243	1.319	1.244	1.317
*r*_C_5_N_1__	1.385	1.354	1.384	1.353	1.384	1.354	1.385	1.355	1.383	1.354	1.385	1.355	1.385	1.356	1.382	1.353	1.383	1.354
*r*_C_7_N_8__	1.350	1.304	1.350	1.305	1.348	1.303	1.347	1.305	1.350	1.307	1.348	1.305	1.348	1.306	1.353	1.305	1.352	1.304
*r*_O_6_H_	—	1.019	—	1.016	—	1.021	—	1.026	—	1.030	—	1.033	—	1.039	—	1.020	—	1.025
*r*_N_8_H_	1.031	—	1.033	—	1.032	—	1.032	—	1.034	—	1.033	—	1.032	—	1.033	—	1.035	—
*δ*, Calc^c^	11.90	14.17	12.09	14.02	12.46	14.47	12.53	14.89	12.13	15.21	12.36	15.38	12.26	15.65	12.82	14.35	12.69	14.62
*δ*, Expt^d^	12.59		12.13		12.81		12.89		12.58		12.75		12.85		12.79		13.22	

a Calculated in the gas phase at the B3LYP/6-311++G(d,p) level. Δ*E*, IPV, and BDE in kcal mol^−1^, dipole moment in Debye, and *r* in Å. Δ*E* is the difference in energy between a given isomer and the amine-one tautomer. For parameters of the imine-one(I, II) tautomeric forms see ESI Table S1 and for properties calculated in chloroform, DMSO, and water for the amine-one and imine-ol forms see ESI Tables S2, S3 and S4, respectively.

b Δ*E* and BDE corrected for ZPE are reported in brackets.

c Calculated chemical shifts (in ppm) for H at O_6_ (imine-ol) and N_8_ (amine-one).

d Data from ref. 21.

The amine-one and imine-ol tautomeric forms are characterized, in respective, by strong intramolecular N_8_–H⋯O_6_ and O_6_–H⋯N_8_ H-bonding (Fig. 3). Relatively weaker C–H⋯O_6_ H-bonds exits in all tautomeric forms and weak C–H⋯N_2_ H-bonding stabilizes all tautomers except the imine-one(II) form, due to protonation of N_2_ (Fig. 3 and S1†). In addition, H-bonding between substituents at the *ortho* position of the phenyl ring of R^2^ (Fig. 2) and N_8_ exists in the three imine tautomers (Fig. 3 and S1†). Besides these σ-type H-bonds, the isopropyl group at C_7_ of compounds 5–7, 9 forms π-type hydrogen bonds (C–H⋯π) with the phenyl group at N_8_ (not shown).

Tables 1 and S1† show that the imine-ol, imine-one(I), and imine-one(II) tautomers are, in respective, 6.5–8.0, 17.0–20.0, and 19.1–23.3 kcal mol^−1^ less stable than the amine-one structures. Considering ZPE corrections decreases these differences in stability by 0.5–1.4 kcal mol^−1^ (Tables 1 and S1†). Note that one should consider approximately half the values reported for compounds 8 and 9 when considering tautomerism in a single Schiff base fragment of the molecule. These data clearly indicate that 4-acylpyrazolone-based Schiff bases exists predominantly in the amine-one tautomeric form and that the imine-one(I, II) forms are unfavourable.

The reported structural properties of the four tautomeric forms present benchmark data to identify the tautomeric form of X-ray structures, especially with the fact that bond distances are almost unaffected by R^1^ and R^2^ substituents (Tables 1 and S1†). We consider the imine-one(I, II) tautomeric forms unfavorable and will mainly focus on differences between the two other forms.

Table 1 shows that the difference between imine-ol and amine-one tautomers in *r*_N_1_N_2__, *r*_N_2_C_3__, and *r*_C_3_C_4__ bond distances is minor (0.004–0.013 Å), which is consistent with the similar order depicted for these bonds in the two tautomers (Fig. 1B). The *r*_C_5_N_1__ bond distance decreases by an average of 0.030 Å on going from the imine-ol (1.382–1.385 Å) to the amine-one (1.353–1.360 Å) tautomer. The decrease of *r*_C_4_C_5__ and *r*_C_7_N_8__ by 0.053–0.058 Å and 0.042–0.048 Å and the increase of *r*_C_4_C_7__ and *r*_C_5_O_6__ by 0.051–0.055 and 0.070–0.077 Å, respectively, reflects a change in bond order of the two bond pairs from single to double and *vice versa* on going from the amine-one to the imine-ol forms (Fig. 1B). While the pyrazolone ring and the phenyl ring at N_1_ are coplanar in the amine-one structures, they possess an interplanar angle of 10–17° in the imine-ol form. This happens to maximize H-bonding between phenyl H and an electron lone pair of O_6_, which occupies SP^2^ orbital in the amine-on isomer but SP^3^ orbital in the imine-ol tautomer.

A number of studies have measured the X-ray crystal structure of different 4-acylpyrazolone Schiff bases and showed that they are characterized by *r*_C_4_C_5__, *r*_C_7_N_8__, *r*_C_4_C_7__, and *r*_C_5_O_6__ bond distances of 1.432–1.448 Å, 1.327–1.344 Å, 1.382–1.400 Å, and 1.245–1.261 Å, respectively.^15,17,18,20^ While *r*_C_4_C_5__ falls between the values observed for the amine-one (1.460–1.464 Å) and imine-ol (1.403–1.407 Å) structures, these distances are more consistent with amine-one rather than imine-ol structures in the solid state (Table 1).^15,17,18,20^

Fig. 3 shows that O_6_–H⋯N_8_ H-bond distances are 0.11–0.19 Å shorter than N_8_–H⋯O_6_ bonds, reflecting that the former is stronger.^26^ The higher stability of the amine-one tautomer is likely due to delocalization of the electrons of the unhybridized 2p orbital of O_6_ over the pyrazolone ring. In addition, the planarity of the pyrazolone and phenyl rings extends this delocalization to the latter ring and maximizes the conjugation among both rings. The much lower stability of the imine-one(I, II) tautomeric forms is attributed to electrostatic repulsion between O_6_ and N_8_ and to breaking the aromaticity of the pyrazolone ring. Additional electrostatic repulsion between H of N_2_ and neighboring phenyl H, results in ∼35° interplanar angle between the pyrazolone and phenyl rings of the imine-one(II) form and makes it the least stable.

It is also seen that the amine-one structures are more polar than the imine-ol tautomers. This is evidenced by a dipole moment of the former being 1.8 to 4.3 times larger than this of the latter (Table 1). The imine-one(II) form is also more polar than the corresponding imine-one(I) tautomer (Table S1†).

### Solvent effects

Tables S2, S3, and S4† report relative stability, IPV, BDE, dipole moment, and selected bond distances of the optimized global minimum amine-one and imine-ol tautomeric forms of compounds 1–9 in chloroform, DMSO, and water, respectively. The total energy of these tautomeric forms in gas phase and in the three solvents is given in Table S5.† Except for IPVs, which are slightly larger in water, DMSO and water have almost identical effects on the calculated properties.

Solvents enlarge the energy difference between the imine-ol and amine-one isomers. So while the imine-ol form is 6.5–8.0 kcal mol^−1^ less stable in gas phase, it is 8.0–9.9 kcal mol^−1^ less stable in chloroform and 8.5–10.5 less stable in both DMSO and water. Again notice that including ZPE corrections lower the difference in stability between the two forms by 0.5–1.9 kcal mol^−1^ (Tables S2–S4†). The effect of solvent on the reported bond distances is minimal and variable. For the amine-one tautomeric form, chloroform increases the *r*_N_1_N_2__, *r*_N_2_C_3__, *r*_C_4_C_7__, *r*_C_5_O_6__, and *r*_C_5_N_1__ distances by 0.001–0.006 Å but decreases *r*_C_3_C_4__, *r*_C_4_C_5__, *r*_C_7_N_8__, and *r*_N_8_H_ distances by 0.002–0.006 Å. The more polar solvents DMSO and water increase and decrease these distances by up to 0.010 Å (Tables S2–S4†). Solvents have relatively smaller effects on the imine-ol bonds except for the *r*_O_6_H_ distance which is increased by 0.001–0.007 Å in chloroform and by 0.002–0.030 Å in DMSO or water (Tables S2–S4†).

The dipole moment of both tautomeric forms increases with solvent polarity, albeit to a larger extent for the amine-one structure. So while going from gas phase to chloroform increases the dipole moment of the amine-one form by 24–31%, it increases this of the imine-ol form by 4–23% (Table 1*vs.* S2†). Going from chloroform to DMSO increases the dipole moment of the two forms by a maximum of 11% and 9% but going from DMSO to water alters the dipole moment of either form by <2% (Tables S2–S4†). The increasing stability of the amine-one relative to the imine-ol form as solvent polarity increases can thus be attributed to the higher increase in polarity of the former; the enlarged difference in polarity translates into enlarged difference in stability.

Compared to gas phase, IPVs of both amine-one and imine-ol tautomeric forms are lowered by 10–17, 7–15, and 6–13 kcal mol^−1^ in chloroform, DMSO and water, respectively. This decrease in IPV is due to the higher stability of the radical cations in solvents as compared to gas phase.^6^

Solvents affect BDEs by a maximum of 3.5 kcal mol^−1^ and the directionality of this change varies between compounds. For example while DMSO increases the BDE of the amine-one form of compound 1 by 1.0 kcal mol^−1^, it decreases this of compound 4 by 3.4 kcal mol^−1^ and does not influence this of compound 3 (Table 1*vs.* S3†). ZPE-corrected BDEs are 6.8–9.3 kcal mol^−1^ lower than the uncorrected values.

### Energy barriers for amine-one to imine-ol interconversions

**Fig. 4:**
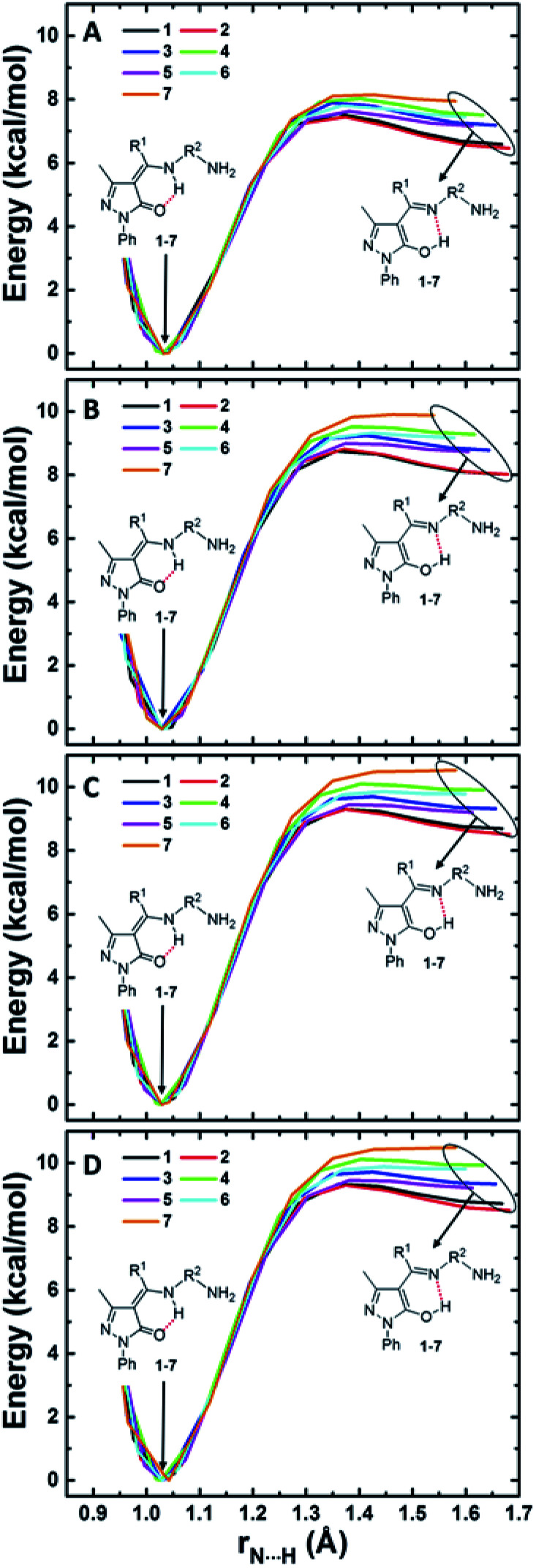
Intramolecular H-transfer in pyrazolone Schiff bases 1–7 in gas phase (A), chloroform (B), DMSO (C), and water (D).

Since O_6_ and N_8_ are H-bonded in both amine-one and imine-ol forms, H transfer from one atom to the other results in interconversion of the two tautomeric form. The energy barriers associated with these transfer provide a measure for the relative rate of the two interconversions. We show in Fig. 4 the change in energy accompanying H atom transfer between the two atoms in compounds 1–7 in gas phase and in the three considered solvents. Besides reflecting the higher stability of the amine-one form, Fig. 4 shows that the energy barrier for converting the imine-ol to the amine-one form is 0.2–1.0 kcal mol^−1^ in gas phase and decreases to 0.0–0.8 kcal mol^−1^ in presence of solvents. This is showing that the imine-ol tautomeric form is not only less stable, but also its conversion to the amine-one structure is almost barrierless. This makes formation of the amine-one form both thermodynamically and kinetically favourable.

Parmar *et al.* measured the ^1^H NMR spectra of compounds 2 and 6 in DMSO and of the other compounds in chloroform and observed singlet peaks at *δ* = 12.13–13.22 ppm which they assigned to H of the imine-ol tautomeric form.^21^ Our calculations show that the chemical shift for H at N_8_ (amine-one), O_6_ (imine-ol), C_4_ (imine-one(I)), and N_2_ (Imine-one(II)) falls in the ranges 11.9–12.9, 14.0–15.7, 3.97–4.62, and 5.03–5.28 ppm, respectively, (Tables 1 and S1†). The experimental chemical shifts fall in the first range, indicating that the compounds adopt the amine-one rather than any of the three imine forms in solution. This finding is supported by the fact that this family of compounds exclusively exist in the amine-one form in the solid state.^15–18,20^ It is also in agreement with experimental results on other pyrazolone Schiff bases in chloroform.^15,17,19^ For example Jadeja *et al.* recorded the ^1^H NMR spectra of seven structurally similar Schiff bases and reported singlet peaks at 12.96–13.12 ppm, which were assigned to H of the amine-one structures.^15^ Amarasekara *et al.* observed peaks at 11.50–12.00 ppm in the ^1^H NMR spectra of five pyrazolone Schiff bases and again assigned them to the amine-one tautomers.^17^ More recently, Listkowski *et al.* showed that the ^1^H NMR spectra of three acylpyrazolone Schiff bases containing methylene group next to N_8_ display triplet peaks at 11.39–11.62 ppm, confirming the existence of the compounds in the amine-one isomeric form.^19^ It could thus be concluded that 4-acylpyrazolone Schiff bases exist in the amine-one tautomeric form in the gas and solid phases and in their solutions. Note that the error in the calculated *δ* of the amine-one structures is only 0.2–5.5% (Table 1), highlighting the reliability of the employed model chemistry.

### Antioxidant activity of Schiff bases 1–9

Since all nine compounds adopt the amine-one tautomeric form, BDE and IPV calculated for this form will be considered to compare the antioxidant power of the compounds. As seen from Table 1, BDEs of compounds 1–9 present 51–62% of the calculated IPV. Solvents lower IPV, yet BDEs still present 55–67% of IPVs (Tables S2–S4†). This indicates that the H-atom transfer mechanism is energetically more favorable than the sequential electron transfer proton transfer mechanism when these Schiff bases interact as antioxidants with free radicals.^6^

Parmar *et al.* experimentally measured the ferric reducing antioxidant power (FRAP) of compounds 1–9 and showed that compounds 4 and 1 are the strongest and weakest antioxidants. The power of the compounds to reduce Fe(iii) to Fe(ii) is measured during these experiments,^21^ and thus IPVs rather than BDEs should be considered to compare the antioxidant activity. Interestingly, the data in Tables S2–S4† show that compounds 1 and 4 possess the highest and lowest IPVs, which is consistent with their observed lowest and highest FRAP, respectively. It should be noted that gas-phase calculations predict compound 8 to be the most efficient antioxidant, indicating that gas-phase calculations of IPV may not be proper for comparing antioxidant activities.

Following 4, PCM calculations suggest that compounds 7, 8, and 9 possess low IPV and in fact these compounds possess the highest FRAP after 4.^21^ It is surprising however that the experimental FRAP of 9 is much higher than that of 8,^21^ as calculations suggest similar or slightly larger FRAP for 8 (Tables S2–S4†).

An analysis of the Mulliken atomic spin densities of the radicals formed upon N–H bond cleavage of the amine-one structures shows that the radical density is centered mainly at N_8_, C_7_, O_6_, C_4_, C_3_, and N_2_ atoms and to a smaller extent at the *ortho* and *para* C atoms of the phenyl ring at N_8_. The presence of the NH_2_ group at these *ortho* or *para* positions in compounds 1, 2, 4, 5, and 7 extends the radical delocalization to the amine N atom^6^ and results in their observed lower BDE, especially in DMSO and water (Tables S3 and S4†). Although the radical spin density is larger at the *para* position, *ortho* NH_2_ stabilize the radical *via* H-bonding with N_8_. Note that negligible radical spin density is observed at the phenyl rings at C_7_ and N_1_.

Analysis of the atomic spin densities of radical cations formed on ionization of the amine-one tautomer show that the radical is centered on the pyrazolone moiety and on the *ortho* and *para* C atoms of the phenyl ring of N_1_ but not on the phenyl rings at C_7_ or N_8_. The fact that the radical spin density at *para* C (0.11–0.20 e) is about twice this at *ortho* C (0.05–0.09 e), indicates that substituents that extend the radical density delocalization would more efficiently increase the FRAP when placed at the *para* position.^6^ This is confirmed by calculating the IPV of two amine-one derivatives of 4 generated by substituting *ortho* or *para* H of the phenyl ring at N_1_ by OH. Note that the OH at the *ortho* position adopts a *cis* conformation relative to N_2_ (structures not shown). Calculations at the B3LYP/6-311++G(d,p) level in gas phase and in presence of the solvents show that *ortho* OH lowers the IPV by a maximum of 0.7 kcal mol^−1^ while *para* OH decreases the IPV by 2.0–3.6 kcal mol^−1^. Interestingly the two derivatives adopt the exact N–H BDEs calculated for the amine-one tautomer of 4, confirming that the phenyl ring at N_1_ does not contribute to stabilizing radicals generated from N–H bond cleavage.

## Conclusions

Tautomerism in 4-acylpyrazolone-based Schiff bases results in four possible isomeric structures. However, only the amine-one and imine-ol tautomers seem energetically plausible, with the first being 6.5–8.0 kcal mol^−1^ more stable in the gas phase and up to 11 kcal mol^−1^ more stable in solvents. H transfer from azomethine N to pyrazolone O is almost barrierless and thus the amine-one tautomeric form is kinetically and thermodynamically favorable. It is thus evident that these compounds maintain the amine-one form in gas phase and in solutions. It is also the form present in the solid phase, as evidenced from crystal structure measurements.^15–18,20^

The limitation of the B3LYP functional in describing dispersion-like interactions^27^ does not alter our findings, as geometry optimization and relaxed scans of compounds 1–4 in gas phase and in DMSO using the M06-2X functional^28^ gave consistent results. M062X/6-311++G(d,p) calculations show 4.0–4.9 and 6.2–7.5 kcal mol^−1^ more stable amine-one tautomer in gas phase and in DMSO, respectively, and show energy barrier for imine-ol to amine-one conversion of 1.6–2.0 kcal mol^−1^ in gas phase and of 1.0–1.6 kcal mol^−1^ in DMSO.

Differentiating between amine-one and imine-ol tautomeric forms based on ^1^H NMR data is reported to be not possible.^15^ Our calculations show however that H of the two forms adopt non-overlapping ranges of chemical shift with this of the amine-one (*δ* = 11.9–12.9 ppm) being lower than the imine-ol form (*δ* = 14.0–15.7 ppm). These data present reference values for identifying the tautomeric form from ^1^H NMR measurements. The reported bond distances for a given tautomeric form of compounds 1–9 are similar and change very little in solvents (Tables 1, S2–S4†). They thus present a valuable benchmark data for identifying the exact tautomeric form for structures obtained from X-ray measurements.

It is found that BDEs are about half the IPV and thus transferring an H atom is energetically easier than transferring an electron to free radicals. This makes the H-atom transfer mechanism more favorable compared to the sequential electron transfer proton transfer mechanism in antioxidant-free radical interactions.^6^

IPV measure the energy required to remove an electron from the molecule and hence indirectly measure its FRAP. It is found that the FRAP of the studied compounds is inversely proportional with their solvent-calculated IPV. Calculations of IPV in presence of solvents can thus be used as a simple tool to predict and compare the antioxidant efficiency of compounds.

The distribution of the radical spin density, in radicals generated from N–H bond cleavage or from electron loss, over the pyrazolone ring indicate the importance of this moiety in stabilizing these radical species and hence in the antioxidant activity of the molecules.

The structure–antioxidant relationship observed for these compounds suggests that the FRAP of 4 could be increased by including substituents such as OH, NH_2_, NO_2_, CN, and π-conjugated systems at the *para* position of phenyl group at N_1_.

## Conflicts of interest

There are no conflicts to declare.

## Supplementary Material

RA-008-C8RA05987J-s001
