# Supplementary material for: Tautomerism and antioxidant activity of some 4-acylpyrazolone-based Schiff bases: a theoretical study
Source: RSC Adv. 2018 Sep 3;8(54):30842–50. doi: 10.1039/c8ra05987j (PMC9085507; doi:10.1039/c8ra05987j)
Supplement: RA-008-C8RA05987J-s001 [file RA-008-C8RA05987J-s001.pdf]

Electronic Supplementary Material (ESI) for RSC Advances.
This journal is © The Royal Society of Chemistry 2018

Supporting Information for:

Tautomerism and antioxidant activity of some 4-acylpyrazolone-based Schiff Bases: a theoretical study

Esam A. Orabi[1,†,*]

[1] Department of Chemistry, Faculty of Science, Assiut University, Assiut 71516, Egypt

[†] Current address: Center for Research in Molecular Modeling (CERMM) and Department of Chemistry and Biochemistry, Concordia University, 7141 Sherbrooke Street West, Montréal, Québec H4B 1R6, Canada

* E-mail: e_orabi@live.concordia.ca and orabiesam@gmail.com

Tel.: +1-514-848-2424, extension 5835; Fax: +1-514-848-2868

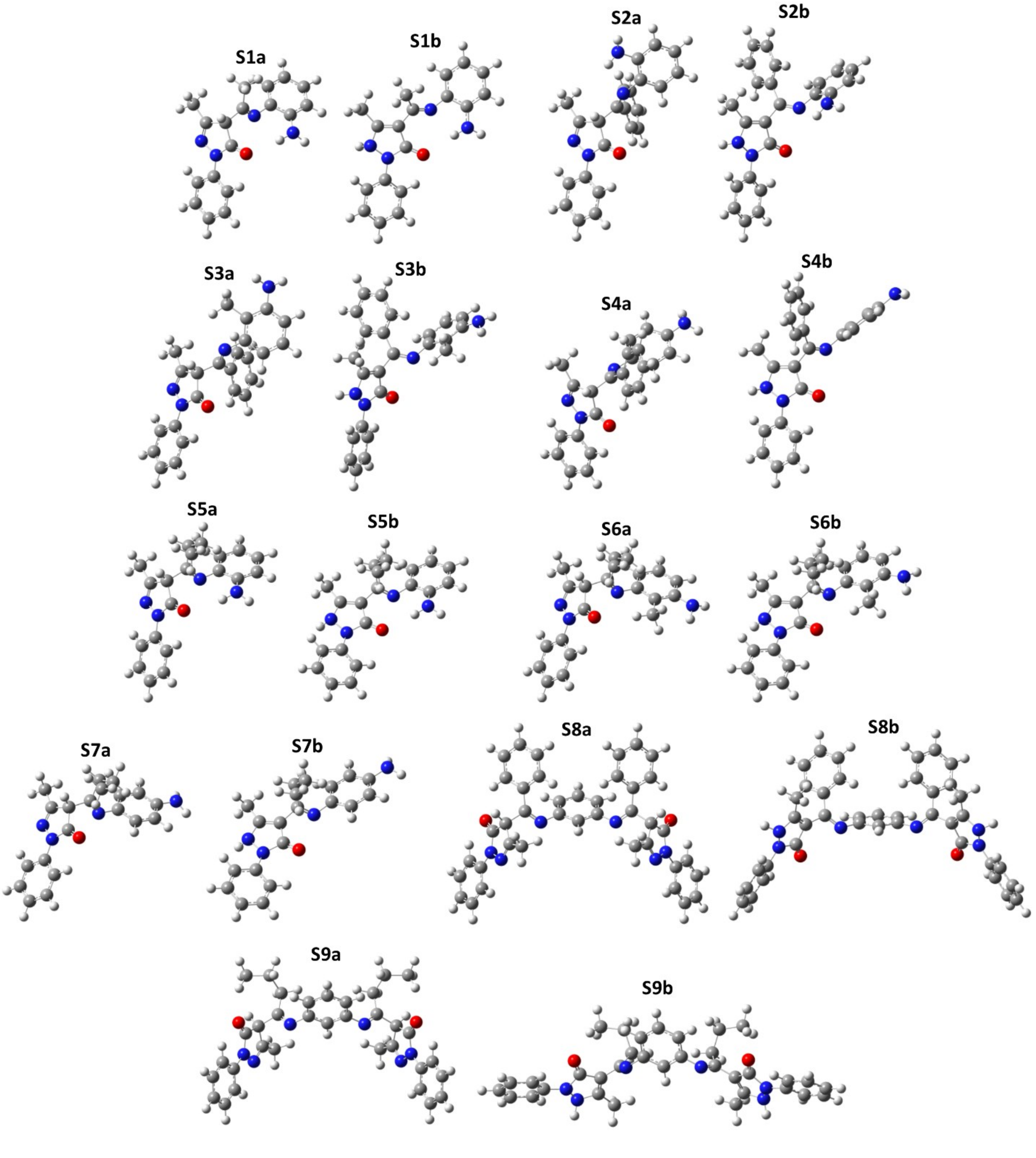

Figure S1. Optimized gas-phase geometry of the *global minimum* conformers of the imine-one(I) (a) and imine-one(II) (b) tautomeric forms of pyrazolone Schiff bases **1–9** at the B3LYP/6-311++G(d,p) level of theory. Atom color code: H (white), C (gray), N (blue), and O (red). For clarity of the structures, intramolecular H-bonds are not shown. The atomic coordinates are provided below.

Table S1. Properties of imine-one(I) and imine-one(II) tautomers of compounds **1–9** in gas phase [a]

Property	**1**		**2**		**3**		**4**		**5**		**6**		**7**		**8**		**9**	
	imine-one(I)	imine-one(II)	imine-one(I)	imine-one(II)	imine-one(I)	imine-one(II)	imine-one(I)	imine-one(II)	imine-one(I)	imine-one(II)	imine-one(I)	imine-one(II)	imine-one(I)	imine-one(II)	imine-one(I)	imine-one(II)	imine-one(I)	imine-one(II)
ΔE [b]	17.9 (16.6)	19.1 (18.3)	17.0 (16.2)	19.9 (19.2)	18.4 (17.2)	21.6 (20.6)	17.5 (16.5)	22.5 (21.6)	17.5 (16.3)	19.1 (18.3)	18.6 (17.3)	21.4 (20.4)	20.0 (18.6)	23.3 (22.2)	33.7 (31.9)	43.5 (41.7)	35.5 (33.2)	42.2 (40.3)
IPV	163.2	154.3	164.0	155.4	163.7	158.0	159.9	153.3	162.7	154.7	163.6	160.3	160.3	155.8	163.3	155.4	163.1	158.5
BDE [b]	75.1 (67.3)	73.8 (65.5)	74.0 (65.8)	71.2 (62.8)	77.9 (69.9)	74.8 (66.5)	78.7 70.4)	73.7 (65.3)	76.3 (68.3)	74.7 (66.4)	78.8 (70.8)	76.0 (67.7)	76.5 (68.9)	73.2 (65.3)	62.0 (54.8)	52.3 (45.0)	60.7 (53.9)	54.0 (46.8)
Dipole	3.11	5.88	3.22	5.85	3.86	5.26	3.74	4.50	3.02	5.71	2.86	4.19	4.15	4.77	6.32	10.75	7.19	10.73
$r_{N_1N_2}$	1.400	1.400	1.402	1.400	1.402	1.403	1.405	1.403	1.401	1.402	1.401	1.403	1.401	1.403	1.404	1.403	1.401	1.403
$r_{N_2C_3}$	1.281	1.389	1.282	1.385	1.282	1.390	1.281	1.391	1.281	1.391	1.281	1.393	1.281	1.393	1.281	1.390	1.281	1.393
$r_{C_3C_4}$	1.512	1.363	1.510	1.365	1.509	1.360	1.507	1.360	1.511	1.360	1.510	1.358	1.510	1.357	1.508	1.359	1.510	1.357
$r_{C_4C_5}$	1.536	1.477	1.538	1.475	1.538	1.474	1.541	1.473	1.534	1.474	1.535	1.472	1.536	1.473	1.542	1.473	1.538	1.472
$r_{C_4C_7}$	1.528	1.474	1.523	1.478	1.525	1.484	1.532	1.484	1.529	1.478	1.531	1.483	1.532	1.484	1.532	1.484	1.531	1.485
$r_{C_5O_6}$	1.212	1.218	1.213	1.214	1.213	1.215	1.215	1.216	1.214	1.219	1.213	1.218	1.212	1.216	1.215	1.215	1.213	1.216
$r_{C_5N_1}$	1.387	1.414	1.388	1.420	1.388	1.416	1.383	1.416	1.386	1.411	1.386	1.414	1.387	1.415	1.382	1.416	1.385	1.414
$r_{C_7N_8}$	1.272	1.279	1.279	1.283	1.276	1.279	1.277	1.280	1.272	1.277	1.271	1.276	1.272	1.276	1.276	1.278	1.272	1.275
r_{C_4H}	1.097	–	1.096	–	1.096	–	1.096	–	1.095	–	1.095	–	1.095	–	1.095	–	1.094	–
r_{N_2H}	–	1.017	–	1.017	–	1.017	–	1.017	–	1.017	–	1.017	–	1.018	–	1.017	–	1.018
δ [c]	3.97	5.09	4.54	5.28	4.62	5.12	4.35	5.05	4.17	5.13	4.15	5.09	4.10	5.03	4.41	5.11	4.12	5.09

[a] Calculated in the gas phase at the B3LYP/6-311++G(d,p) level. ΔE, IPV, and BDE in kcal/mol, dipole moment in debye, and r in Å. ΔE is the difference in energy between a given isomer and the amine-one tautomer. [b] ΔE and BDE corrected for ZPE are reported in brackets. [c] Calculated chemical shifts (in ppm) for H at C_4 (imine-one(I)) or at N_2 (imine-one(II)).

Table S2. Properties of amine-one and imine-ol tautomers of compounds **1–9** in chloroform [a]

Property	**1**		**2**		**3**		**4**		**5**		**6**		**7**		**8**		**9**	
	amine-one	imine-ol	amine-one	imine-ol	amine-one	imine-ol	amine-one	imine-ol	amine-one	imine-ol	amine-one	imine-ol	amine-one	imine-ol	amine-one	imine-ol	amine-one	imine-ol
ΔE [b]	0.0 (0.0)	8.0 (7.4)	0.0 (0.0)	8.0 (7.4)	0.0 (0.0)	8.8 (7.9)	0.0 (0.0)	9.3 (8.3)	0.0 (0.0)	8.7 (7.8)	0.0 (0.0)	9.2 (8.1)	0.0 (0.0)	9.9 (8.4)	0.0 (0.0)	16.3 (14.7)	0.0 (0.0)	16.2 (14.1)
IPV [c]	148.7	146.9	147.9	145.9	146.2	148.0	143.0	140.9	147.9	147.0	146.7	148.9	144.9	143.3	144.9	149.4	145.5	152.7
BDE [b]	93.9 (85.1)	85.8 (77.7)	92.3 (83.4)	84.3 (76.0)	96.5 (87.3)	87.7 (79.4)	94.6 (85.6)	85.3 (77.3)	94.2 (85.3)	85.5 (77.5)	97.9 (88.6)	88.7 (80.5)	95.2 (86.2)	85.3 (77.8)	95.7 (86.8)	79.5 (72.0)	96.4 (87.2)	80.2 (73.1)
Dipole	7.41	1.60	7.26	1.76	8.28	3.29	9.67	4.87	6.87	1.99	8.16	3.52	9.52	4.67	14.32	6.27	9.16	4.91
$r_{N_1N_2}$	1.394	1.387	1.395	1.388	1.395	1.388	1.395	1.388	1.393	1.387	1.392	1.387	1.392	1.387	1.395	1.388	1.393	1.386
$r_{N_2C_3}$	1.307	1.321	1.307	1.321	1.308	1.321	1.308	1.321	1.308	1.320	1.308	1.321	1.308	1.321	1.306	1.320	1.307	1.320
$r_{C_3C_4}$	1.444	1.432	1.444	1.434	1.444	1.433	1.443	1.433	1.445	1.436	1.445	1.435	1.445	1.434	1.446	1.434	1.446	1.435
$r_{C_4C_5}$	1.455	1.402	1.456	1.403	1.456	1.404	1.455	1.404	1.456	1.406	1.456	1.406	1.455	1.407	1.459	1.405	1.458	1.406
$r_{C_4C_7}$	1.398	1.450	1.396	1.448	1.398	1.449	1.400	1.449	1.401	1.449	1.403	1.451	1.403	1.449	1.395	1.447	1.398	1.450
$r_{C_5O_6}$	1.248	1.322	1.247	1.322	1.247	1.320	1.248	1.319	1.249	1.318	1.249	1.317	1.249	1.314	1.247	1.319	1.248	1.318
$r_{C_5N_1}$	1.387	1.353	1.386	1.352	1.386	1.353	1.387	1.354	1.386	1.353	1.386	1.354	1.387	1.355	1.384	1.352	1.385	1.353
$r_{C_7N_8}$	1.344	1.304	1.345	1.305	1.343	1.302	1.342	1.305	1.346	1.307	1.345	1.305	1.342	1.306	1.348	1.304	1.347	1.304
r_{O_6H}	–	1.021	–	1.017	–	1.025	–	1.032	–	1.033	–	1.040	–	1.054	–	1.023	–	1.032
r_{N_8H}	1.028	–	1.029	–	1.028	–	1.029	–	1.031	–	1.030	–	1.029	–	1.030	–	1.031	–

[a] Calculated at the B3LYP/6-311++G(d,p) level in chloroform using the PCM model. ΔE, IPV, and BDE in kcal/mol, dipole moment in debye, and r in Å. ΔE is the difference in energy between a given isomer and the amine-one tautomer. [b] ΔE and BDE corrected for ZPE are reported in brackets. [c] IPV is calculated using the non-equilibrium polarizable continuum model.

Table S3. Properties of amine-one and imine-ol tautomers of compounds **1–9** in DMSO [a]

Property	**1**		**2**		**3**		**4**		**5**		**6**		**7**		**8**		**9**	
	amine-one	imine-ol	amine-one	imine-ol	amine-one	imine-ol	amine-one	imine-ol	amine-one	imine-ol	amine-one	imine-ol	amine-one	imine-ol	amine-one	imine-ol	amine-one	imine-ol
ΔE [b]	0.0 (0.0)	8.7 (8.1)	0.0 (0.0)	8.5 (8.0)	0.0 (0.0)	9.3 (8.3)	0.0 (0.0)	9.9 (8.8)	0.0 (0.0)	9.2 (8.3)	0.0 (0.0)	9.8 (8.5)	0.0 (0.0)	10.5 (8.6)	0.0 (0.0)	17.3 (15.7)	0.0 (0.0)	17.6 (15.4)
IPV [c]	150.6	149.1	150.5	148.6	149.3	150.8	145.9	143.6	150.5	149.2	149.7	151.3	147.6	145.4	148.0	152.5	148.1	155.2
BDE [b]	94.0 (85.4)	85.3 (77.3)	92.6 (84.0)	84.1 (76.0)	96.3 (87.1)	87.0 (78.8)	92.8 (84.1)	82.9 (75.3)	93.5 (84.5)	84.3 (76.2)	98.8 (89.6)	89.1 (81.1)	93.6 (84.5)	83.1 (75.9)	95.6 (86.6)	78.3 (71.0)	96.2 (87.1)	78.6 (71.7)
Dipole	7.56	1.67	7.58	1.65	8.76	3.36	9.78	5.01	7.38	2.03	8.68	3.69	10.19	5.05	15.45	6.40	10.19	5.37
$r_{N_1N_2}$	1.395	1.387	1.396	1.388	1.397	1.388	1.395	1.388	1.394	1.387	1.393	1.387	1.393	1.388	1.396	1.388	1.393	1.387
$r_{N_2C_3}$	1.309	1.322	1.308	1.322	1.309	1.322	1.309	1.322	1.309	1.322	1.310	1.322	1.310	1.322	1.308	1.322	1.309	1.322
$r_{C_3C_4}$	1.441	1.432	1.443	1.433	1.443	1.433	1.442	1.433	1.444	1.435	1.444	1.434	1.444	1.434	1.444	1.433	1.445	1.435
$r_{C_4C_5}$	1.450	1.401	1.454	1.403	1.454	1.403	1.453	1.404	1.454	1.406	1.454	1.406	1.453	1.407	1.456	1.404	1.456	1.406
$r_{C_4C_7}$	1.401	1.450	1.397	1.448	1.400	1.449	1.402	1.450	1.403	1.450	1.405	1.451	1.406	1.449	1.397	1.447	1.402	1.451
$r_{C_5O_6}$	1.250	1.322	1.248	1.322	1.249	1.320	1.249	1.319	1.250	1.318	1.250	1.317	1.251	1.313	1.248	1.320	1.250	1.318
$r_{C_5N_1}$	1.388	1.353	1.386	1.351	1.387	1.352	1.387	1.353	1.387	1.352	1.386	1.354	1.388	1.355	1.385	1.352	1.385	1.352
$r_{C_7N_8}$	1.341	1.304	1.343	1.304	1.341	1.302	1.340	1.305	1.344	1.307	1.341	1.304	1.340	1.307	1.346	1.304	1.344	1.304
r_{O_6H}	–	1.022	–	1.018	–	1.026	–	1.034	–	1.034	–	1.044	–	1.068	–	1.024	–	1.035
r_{N_8H}	1.029	–	1.028	–	1.027	–	1.027	–	1.030	–	1.029	–	1.028	–	1.029	–	1.030	–

[a] Calculated at the B3LYP/6-311++G(d,p) level in DMSO using the PCM model. ΔE, IPV, and BDE in kcal/mol, dipole moment in debye, and r in Å. ΔE is the difference in energy between a given isomer and the amine-one tautomer. [b] ΔE and BDE corrected for ZPE are reported in brackets. [c] IPV is calculated using the non-equilibrium polarizable continuum model.

Table S4. Properties of amine-one and imine-ol tautomers of compounds **1–9** in water [a]

Property	**1**		**2**		**3**		**4**		**5**		**6**		**7**		**8**		**9**	
	amine-one	imine-ol	amine-one	imine-ol	amine-one	imine-ol	amine-one	imine-ol	amine-one	imine-ol	amine-one	imine-ol	amine-one	imine-ol	amine-one	imine-ol	amine-one	imine-ol
ΔE [b]	0.0 (0.0)	8.7 (8.1)	0.0 (0.0)	8.5 (7.9)	0.0 (0.0)	9.3 (8.4)	0.0 (0.0)	9.9 (8.9)	0.0 (0.0)	9.3 (8.3)	0.0 (0.0)	9.8 (8.5)	0.0 (0.0)	10.5 (8.6)	0.0 (0.0)	17.5 (15.9)	0.0 (0.0)	17.7 (15.5)
IPV [c]	152.4	151.0	152.1	150.3	150.8	152.6	147.3	145.3	152.1	150.9	151.2	153.0	149.1	147.2	149.0	153.6	149.2	156.1
BDE [b]	94.0 (85.4)	85.3 (77.3)	92.6 (83.9)	84.1 (76.0)	96.3 (87.2)	87.0 (78.7)	92.7 (84.1)	82.7 (75.2)	93.5 (84.4)	84.2 (76.1)	98.8 (89.6)	89.0 (81.1)	93.5 (84.4)	83.0 (75.9)	95.6 (86.7)	78.2 (70.8)	96.2 (87.2)	78.5 (71.7)
Dipole	7.72	1.68	7.69	1.64	8.74	3.35	9.80	5.02	7.42	2.04	8.70	3.71	10.11	5.08	15.11	6.38	10.28	5.40
$r_{N_1N_2}$	1.395	1.387	1.396	1.388	1.395	1.388	1.394	1.388	1.394	1.387	1.393	1.387	1.393	1.388	1.395	1.388	1.393	1.387
$r_{N_2C_3}$	1.309	1.323	1.308	1.322	1.309	1.322	1.310	1.322	1.309	1.322	1.310	1.322	1.310	1.322	1.308	1.322	1.309	1.322
$r_{C_3C_4}$	1.441	1.432	1.443	1.433	1.443	1.433	1.443	1.433	1.444	1.435	1.444	1.434	1.444	1.434	1.445	1.433	1.446	1.435
$r_{C_4C_5}$	1.450	1.401	1.454	1.403	1.454	1.403	1.453	1.404	1.454	1.406	1.454	1.406	1.453	1.408	1.456	1.404	1.455	1.406
$r_{C_4C_7}$	1.401	1.450	1.398	1.448	1.400	1.449	1.402	1.450	1.404	1.450	1.405	1.451	1.406	1.449	1.397	1.447	1.402	1.451
$r_{C_5O_6}$	1.250	1.322	1.248	1.322	1.249	1.321	1.249	1.319	1.250	1.318	1.250	1.317	1.251	1.313	1.248	1.320	1.250	1.318
$r_{C_5N_1}$	1.389	1.353	1.387	1.351	1.386	1.352	1.387	1.353	1.387	1.352	1.386	1.353	1.387	1.355	1.384	1.351	1.385	1.352
$r_{C_7N_8}$	1.340	1.304	1.343	1.304	1.341	1.302	1.340	1.305	1.344	1.307	1.341	1.305	1.340	1.307	1.346	1.304	1.344	1.304
r_{O_6H}	–	1.022	–	1.017	–	1.025	–	1.034	–	1.034	–	1.044	–	1.069	–	1.024	–	1.035

r_{N_8H}	1.028	–	1.028	–	1.028	–	1.027	–	1.030	–	1.029	–	1.028	–	1.028	–	1.030	–

[a] Calculated at the B3LYP/6-311++G(d,p) level in water using the PCM model. ΔE, IPV, and BDE in kcal/mol, dipole moment in debye, and r in Å. ΔE is the difference in energy between a given isomer and the amine-one tautomer. [b] ΔE and BDE corrected for ZPE are reported in brackets. [c] IPV is calculated using the non-equilibrium polarizable continuum model.

Table S5. Total energy (a.u.) of amine-one, imine-ol, imine-one(I), and imine-one(II) tautomeric forms of compounds **1–9** calculated at the B3LYP/6-311++G(d,p) level of theory in gas phase, chloroform, DMSO, and water.[a]

Compound	Tautomer	Gas phase	Chloroform	DMSO	Water
**1**	amine-one	-991.260472	-991.270863	-991.275622	-991.275878
	imine-ol	-991.249972	-991.258072	-991.261778	-991.261984
	imine-one(I)	-991.232011			
	imine-one(II)	-991.229999			
**2**	amine-one	-1183.037979	-1183.048936	-1183.053786	-1183.054018
	imine-ol	-1183.027678	-1183.036158	-1183.040230	-1183.040465
	imine-one(I)	-1183.010818			
	imine-one(II)	-1183.006260			
**3**	amine-one	-1222.363286	-1222.375007	-1222.380212	-1222.380492
	imine-ol	-1222.351833	-1222.361007	-1222.365369	-1222.365652
	imine-one(I)	-1222.333982			
	imine-one(II)	-1222.328915			
**4**	amine-one	-1183.037238	-1183.049628	-1183.054999	-1183.055282
	imine-ol	-1183.025276	-1183.034838	-1183.039223	-1183.039486
	imine-one(I)	-1183.009405			
	imine-one(II)	-1183.001346			
**5**	amine-one	-1069.905345	-1069.915408	-1069.919836	-1069.920061
	imine-ol	-1069.893926	-1069.901481	-1069.905124	-1069.905283
	imine-one(I)	-1069.877500			
	imine-one(II)	-1069.874919			
**6**	amine-one	-1109.230356	-1109.241267	-1109.246099	-1109.246358
	imine-ol	-1109.218448	-1109.226639	-1109.230521	-1109.230697
	imine-one(I)	-1109.200782			
	imine-one(II)	-1109.196291			
**7**	amine-one	-1069.903733	-1069.915484	-1069.920601	-1069.920867
	imine-ol	-1069.891051	-1069.899730	-1069.903863	-1069.904077
	imine-one(I)	-1069.871914			
	imine-one(II)	-1069.866619			
**8**	amine-one	-2023.008583	-2023.025261	-2023.032621	-2023.033190
	imine-ol	-2022.987531	-2022.999320	-2023.005067	-2023.005368
	imine-one(I)	-2022.954805			
	imine-one(II)	-2022.939258			
**9**	amine-one	-1796.739780	-1796.755530	-1796.762846	-1796.763327
	imine-ol	-1796.719701	-1796.729684	-1796.734786	-1796.735087
	imine-one(I)	-1796.683142			
	imine-one(II)	-1796.672512			

[a] Only the amine-one and imine-ol tautomeric forms are studied in chloroform, DMSO, and water.

Atomic coordinates for the amine-one and imine-ol tautomeric forms presented in Figure 1

Structure 1a

C	-1.083994000	2.166411000	-0.152974000
C	-0.162979000	1.051485000	-0.147007000
C	-1.014062000	-0.133537000	-0.085918000
N	-2.306908000	0.363832000	-0.061787000
N	-2.320743000	1.754947000	-0.101415000
C	-3.536741000	-0.336707000	-0.001556000
C	-3.572258000	-1.738044000	0.032879000
C	-4.735415000	0.389456000	0.023526000
C	-4.801210000	-2.391221000	0.092137000
H	-2.649358000	-2.296983000	0.012807000
C	-5.951554000	-0.282725000	0.082624000
H	-4.697931000	1.468685000	-0.004070000
C	-5.996020000	-1.675729000	0.117581000
H	-4.816964000	-3.475354000	0.118065000
H	-6.871391000	0.291589000	0.101250000
H	-6.946765000	-2.194290000	0.163487000
O	-0.669857000	-1.329083000	-0.060160000
H	1.101095000	-1.045958000	-0.155116000
C	-0.828959000	3.641850000	-0.204515000
H	-0.223966000	3.980016000	0.641905000
H	-0.312931000	3.932645000	-1.124636000
H	-1.786813000	4.161856000	-0.171815000
C	1.224852000	0.955723000	-0.184938000
C	2.152639000	2.133797000	-0.248721000
H	2.852361000	2.016296000	-1.079961000
H	1.612222000	3.065604000	-0.372479000
H	2.747306000	2.188777000	0.668011000
N	1.783759000	-0.272969000	-0.156225000
C	3.184602000	-0.559589000	-0.206616000
C	3.959107000	-0.463714000	0.966989000
C	3.764972000	-0.988513000	-1.400008000
C	5.325123000	-0.775315000	0.888436000
C	5.118554000	-1.304927000	-1.462467000
H	3.136276000	-1.069094000	-2.279729000
C	5.894909000	-1.188486000	-0.309208000
H	5.936070000	-0.697884000	1.782072000
H	5.559556000	-1.635721000	-2.394599000
H	6.951678000	-1.428857000	-0.339162000
N	3.389540000	-0.015938000	2.157604000
H	2.397603000	-0.187076000	2.251747000
H	3.901883000	-0.251982000	2.994803000

Structure 1b

C	-1.121672000	2.262579000	0.127324000
C	-0.172327000	1.193002000	0.037054000
C	-0.971964000	0.045397000	-0.068169000
N	-2.270124000	0.431082000	-0.043121000
N	-2.358164000	1.808090000	0.081886000
C	-3.460318000	-0.347070000	-0.091240000
C	-3.441075000	-1.674431000	-0.532482000
C	-4.665399000	0.245719000	0.300151000
C	-4.629763000	-2.400086000	-0.568363000
H	-2.514233000	-2.134269000	-0.840483000
C	-5.842821000	-0.492819000	0.252460000
H	-4.662786000	1.275229000	0.628277000
C	-5.833712000	-1.818808000	-0.178443000
H	-4.607777000	-3.428574000	-0.910599000
H	-6.772728000	-0.026090000	0.557312000
H	-6.753916000	-2.390422000	-0.211743000
O	-0.557654000	-1.205279000	-0.169442000
H	0.455854000	-1.104054000	-0.155401000
C	-0.904625000	3.738898000	0.254081000
H	-0.398554000	3.993411000	1.189915000
H	-0.298973000	4.130887000	-0.567175000
H	-1.872192000	4.241265000	0.240933000
C	1.272328000	1.106818000	0.020577000
C	2.096613000	2.368523000	0.088323000
H	2.014207000	2.934932000	-0.844119000
H	1.729448000	3.010851000	0.890459000
H	3.147199000	2.144439000	0.262630000
N	1.778404000	-0.093288000	-0.039264000
C	3.161463000	-0.358286000	-0.170922000
C	3.788247000	-1.136386000	0.828896000
C	3.887826000	0.022013000	-1.302817000
C	5.133656000	-1.490540000	0.663595000
C	5.224021000	-0.339164000	-1.457459000
H	3.381707000	0.586097000	-2.078653000
C	5.844122000	-1.097982000	-0.465824000
H	5.622053000	-2.076314000	1.436095000
H	5.768361000	-0.040365000	-2.345274000
H	6.882780000	-1.390131000	-0.571450000
N	3.087200000	-1.476840000	1.984023000
H	2.081279000	-1.430437000	1.902767000
H	3.410689000	-2.301801000	2.466922000

Structure 2a

C	-1.309400000	1.872051000	0.157898000
C	-0.588211000	0.631772000	-0.026129000
C	-1.623445000	-0.394133000	-0.115194000
N	-2.815195000	0.302775000	-0.023098000
N	-2.597823000	1.667640000	0.157440000

C	-4.144385000	-0.185964000	-0.051120000
C	-4.411974000	-1.546921000	-0.256935000
C	-5.205882000	0.712171000	0.125383000
C	-5.732505000	-1.989521000	-0.282449000
H	-3.594533000	-2.238446000	-0.391971000
C	-6.517006000	0.249042000	0.096015000
H	-4.989332000	1.758917000	0.281363000
C	-6.792099000	-1.102503000	-0.107151000
H	-5.928186000	-3.044179000	-0.442661000
H	-7.328944000	0.954766000	0.233763000
H	-7.815959000	-1.457688000	-0.129073000
O	-1.477269000	-1.625285000	-0.231505000
H	0.310942000	-1.629862000	-0.177528000
C	-0.800796000	3.263853000	0.369855000
H	-0.064102000	3.302010000	1.176135000
H	-0.318874000	3.661472000	-0.526605000
H	-1.642509000	3.908011000	0.626340000
C	0.765117000	0.310592000	-0.054679000
N	1.120162000	-0.990520000	-0.118894000
C	2.440463000	-1.519643000	-0.271641000
C	3.007774000	-2.229494000	0.804842000
C	3.132449000	-1.390266000	-1.474708000
C	4.281669000	-2.792099000	0.631450000
C	4.398571000	-1.944455000	-1.630749000
H	2.661074000	-0.853960000	-2.289815000
C	4.966173000	-2.648833000	-0.568488000
H	4.734181000	-3.336696000	1.453903000
H	4.929041000	-1.837319000	-2.568791000
H	5.949248000	-3.093438000	-0.675327000
N	2.350949000	-2.305135000	2.030604000
H	1.350015000	-2.173992000	2.007262000
H	2.632037000	-3.065902000	2.631125000
C	1.845309000	1.335497000	0.002599000
C	2.696715000	1.399827000	1.111548000
C	2.026349000	2.228880000	-1.057373000
C	3.703391000	2.360167000	1.163641000
H	2.568398000	0.701775000	1.931060000
C	3.044432000	3.178272000	-1.008292000
H	1.375586000	2.172509000	-1.922561000
C	3.880474000	3.249419000	0.104359000
H	4.352456000	2.410453000	2.030369000
H	3.182900000	3.861942000	-1.838140000
H	4.668971000	3.992306000	0.144734000

Structure 2b

C	-1.276622000	1.920046000	0.245944000
C	-0.563139000	0.693289000	0.033145000
C	-1.579988000	-0.264901000	-0.107681000
N	-2.771893000	0.368727000	-0.018308000
N	-2.579177000	1.722122000	0.214719000

C	-4.093772000	-0.153485000	-0.075553000
C	-4.350636000	-1.412353000	-0.628628000
C	-5.145227000	0.624597000	0.419936000
C	-5.659854000	-1.886533000	-0.670096000
H	-3.542600000	-2.013425000	-1.017878000
C	-6.446577000	0.137182000	0.364484000
H	-4.928761000	1.599422000	0.832853000
C	-6.712713000	-1.119976000	-0.176625000
H	-5.852299000	-2.863464000	-1.098975000
H	-7.256482000	0.746071000	0.750214000
H	-7.728555000	-1.495742000	-0.215373000
O	-1.430507000	-1.567494000	-0.273660000
H	-0.423717000	-1.686833000	-0.212777000
C	-0.765613000	3.302076000	0.510149000
H	-0.021625000	3.305601000	1.310045000
H	-0.290842000	3.734161000	-0.373779000
H	-1.602591000	3.938203000	0.799962000
C	0.830703000	0.310183000	-0.006221000
N	1.085919000	-0.969678000	-0.022106000
C	2.382479000	-1.516115000	-0.185580000
C	2.866674000	-2.373924000	0.826264000
C	3.132663000	-1.330238000	-1.349137000
C	4.101267000	-3.008253000	0.641572000
C	4.357337000	-1.969389000	-1.523227000
H	2.735791000	-0.689690000	-2.127946000
C	4.839243000	-2.809840000	-0.520763000
H	4.482023000	-3.658240000	1.423329000
H	4.923380000	-1.817436000	-2.434487000
H	5.789990000	-3.316332000	-0.642985000
N	2.150136000	-2.511009000	2.017326000
H	1.167600000	-2.284313000	1.943392000
H	2.317155000	-3.366998000	2.526134000
C	1.898398000	1.358744000	-0.020493000
C	2.819155000	1.434460000	1.030330000
C	1.994823000	2.262075000	-1.083492000
C	3.814647000	2.407913000	1.020217000
H	2.753935000	0.731292000	1.852846000
C	3.003511000	3.222602000	-1.100772000
H	1.287436000	2.203495000	-1.903531000
C	3.911420000	3.301172000	-0.046010000
H	4.519138000	2.464012000	1.842257000
H	3.078818000	3.909878000	-1.935973000
H	4.692312000	4.053150000	-0.056041000

Structure 3a

C	-1.643018000	1.932169000	0.240320000
C	-0.808378000	0.778988000	-0.016935000
C	-1.742778000	-0.335755000	-0.151149000
N	-2.994859000	0.238652000	-0.016471000
N	-2.906444000	1.605746000	0.235774000

C	-4.271242000	-0.373256000	-0.060393000
C	-4.410851000	-1.740826000	-0.337264000
C	-5.411338000	0.408377000	0.172162000
C	-5.683464000	-2.305831000	-0.376382000
H	-3.532912000	-2.342721000	-0.514964000
C	-6.672676000	-0.175909000	0.127884000
H	-5.293419000	1.461337000	0.382598000
C	-6.820491000	-1.534971000	-0.145564000
H	-5.779835000	-3.364496000	-0.591463000
H	-7.546304000	0.440603000	0.309547000
H	-7.806081000	-1.984949000	-0.178650000
O	-1.483095000	-1.540277000	-0.326847000
H	0.290541000	-1.372425000	-0.221454000
C	-1.272456000	3.354027000	0.528139000
H	-0.530654000	3.420884000	1.327761000
H	-0.848424000	3.850163000	-0.348138000
H	-2.170838000	3.893097000	0.830761000
C	0.571516000	0.591079000	-0.068919000
N	1.042335000	-0.666790000	-0.185688000
C	2.381838000	-1.107965000	-0.399310000
C	2.875092000	-2.148903000	0.405222000
C	3.148079000	-0.556950000	-1.430021000
C	4.172516000	-2.638625000	0.140021000
C	4.433615000	-1.034891000	-1.653429000
H	2.732588000	0.220506000	-2.055639000
C	4.944769000	-2.068638000	-0.880261000
H	5.033800000	-0.612704000	-2.451221000
H	5.944182000	-2.447767000	-1.067884000
C	1.538463000	1.719530000	0.049879000
C	2.385683000	1.801455000	1.160381000
C	1.609511000	2.702885000	-0.941353000
C	3.282258000	2.859395000	1.281301000
H	2.337868000	1.040247000	1.930383000
C	2.518125000	3.752029000	-0.825718000
H	0.960439000	2.637503000	-1.807242000
C	3.352936000	3.834669000	0.287342000
H	3.928113000	2.919726000	2.149766000
H	2.572551000	4.504856000	-1.603901000
H	4.056198000	4.654491000	0.379582000
N	4.712932000	-3.647515000	0.942743000
H	4.058387000	-4.304890000	1.339446000
H	5.527431000	-4.106630000	0.562607000
C	2.078654000	-2.742511000	1.540373000
H	1.192089000	-2.153275000	1.769316000
H	1.736589000	-3.759986000	1.311801000
H	2.686539000	-2.799431000	2.449302000

Structure 3b

C	1.621000000	1.945287000	-0.315184000
C	0.810131000	0.799321000	-0.019736000

C	1.743371000	-0.236355000	0.151672000
N	2.983641000	0.284736000	0.000082000
N	2.902141000	1.636178000	-0.300746000
C	4.256789000	-0.346087000	0.058215000
C	4.413049000	-1.610449000	0.636540000
C	5.365486000	0.329190000	-0.463383000
C	5.678322000	-2.191859000	0.677901000
H	3.561290000	-2.133443000	1.044468000
C	6.621649000	-0.265019000	-0.408250000
H	5.227596000	1.309321000	-0.896728000
C	6.787101000	-1.527972000	0.158839000
H	5.791941000	-3.172378000	1.126378000
H	7.475715000	0.265120000	-0.814570000
H	7.768082000	-1.987170000	0.197413000
O	1.482352000	-1.508103000	0.389530000
H	0.462531000	-1.534930000	0.354867000
C	1.226198000	3.352091000	-0.641219000
H	0.469012000	3.381837000	-1.428136000
H	0.809099000	3.867242000	0.227206000
H	2.109136000	3.897239000	-0.976600000
C	-0.610481000	0.536576000	0.063633000
N	-0.964393000	-0.712283000	0.179932000
C	-2.294920000	-1.151317000	0.392898000
C	-2.855545000	-2.075468000	-0.507712000
C	-2.994332000	-0.755095000	1.540458000
C	-4.145804000	-2.578591000	-0.242164000
C	-4.263266000	-1.266508000	1.785049000
H	-2.533479000	-0.066451000	2.236418000
C	-4.842200000	-2.169146000	0.902699000
H	-4.803732000	-0.963469000	2.674946000
H	-5.834413000	-2.564426000	1.095468000
C	-1.586078000	1.671358000	0.000159000
C	-2.492441000	1.763586000	-1.060995000
C	-1.606396000	2.645705000	1.003179000
C	-3.400089000	2.818140000	-1.120226000
H	-2.485733000	1.010045000	-1.839799000
C	-2.526807000	3.689656000	0.952254000
H	-0.909241000	2.577419000	1.831174000
C	-3.422243000	3.780884000	-0.112322000
H	-4.094109000	2.883831000	-1.950463000
H	-2.543564000	4.432064000	1.742309000
H	-4.134648000	4.596954000	-0.155250000
N	-4.757837000	-3.444343000	-1.156742000
H	-4.139214000	-4.023238000	-1.704883000
H	-5.538697000	-3.968496000	-0.789917000
C	-2.125360000	-2.519272000	-1.750408000
H	-1.160100000	-2.024589000	-1.841509000
H	-1.936565000	-3.600865000	-1.745383000
H	-2.707395000	-2.298735000	-2.652714000

Structure 4a

C	1.606735000	1.843336000	0.094276000
C	0.783858000	0.657896000	0.002774000
C	1.731259000	-0.452598000	-0.036994000
N	2.977851000	0.149665000	-0.011270000
N	2.875095000	1.535307000	0.083115000
C	4.261835000	-0.446885000	-0.032562000
C	4.414324000	-1.836723000	-0.140468000
C	5.396578000	0.371847000	0.051513000
C	5.694096000	-2.386285000	-0.162012000
H	3.540582000	-2.466973000	-0.203762000
C	6.665204000	-0.197673000	0.027938000
H	5.268639000	1.441380000	0.133078000
C	6.825829000	-1.578477000	-0.078438000
H	5.800257000	-3.462424000	-0.245665000
H	7.534445000	0.447658000	0.093691000
H	7.817053000	-2.016827000	-0.096160000
O	1.485933000	-1.672423000	-0.069780000
H	-0.284534000	-1.513174000	0.075898000
C	1.221562000	3.284449000	0.224288000
H	0.752259000	3.664603000	-0.686386000
H	0.512826000	3.438195000	1.041773000
H	2.121385000	3.868593000	0.420197000
C	-0.594715000	0.449155000	0.021057000
N	-1.048079000	-0.819132000	0.050207000
C	-2.375928000	-1.320648000	-0.011613000
C	-2.702238000	-2.410394000	0.802642000
C	-3.343093000	-0.831779000	-0.894358000
C	-3.964252000	-2.986227000	0.751881000
C	-4.610385000	-1.398698000	-0.936695000
H	-3.107512000	-0.013565000	-1.561144000
C	-4.945869000	-2.485357000	-0.115887000
H	-5.347554000	-1.000247000	-1.626201000
C	-1.571050000	1.575902000	0.051569000
C	-1.714023000	2.424006000	-1.050764000
C	-2.353458000	1.792321000	1.191074000
C	-2.629995000	3.473029000	-1.015456000
H	-1.113161000	2.254746000	-1.937232000
C	-3.257842000	2.850088000	1.229276000
H	-2.249243000	1.135313000	2.046765000
C	-3.400474000	3.690293000	0.125568000
H	-2.738879000	4.121541000	-1.877381000
H	-3.853035000	3.016055000	2.119907000
H	-4.108825000	4.510400000	0.155023000
H	-1.957158000	-2.807237000	1.483427000
H	-4.194464000	-3.827421000	1.397388000
N	-6.235068000	-3.018946000	-0.124139000
H	-6.774742000	-2.855376000	-0.961124000
H	-6.322153000	-3.967587000	0.209096000

Structure 4b

C	-1.592879000	1.886003000	-0.122727000
C	-0.779325000	0.708651000	-0.022272000
C	-1.713382000	-0.339177000	0.043452000
N	-2.955277000	0.201114000	0.015982000
N	-2.874962000	1.580971000	-0.097357000
C	-4.229895000	-0.427993000	0.039830000
C	-4.370095000	-1.768568000	0.415840000
C	-5.357269000	0.324218000	-0.308207000
C	-5.637776000	-2.346043000	0.430273000
H	-3.504015000	-2.352266000	0.688739000
C	-6.615102000	-0.268407000	-0.283055000
H	-5.231494000	1.360564000	-0.586666000
C	-6.764894000	-1.605437000	0.082985000
H	-5.738307000	-3.385644000	0.721236000
H	-7.483146000	0.321968000	-0.554363000
H	-7.747402000	-2.062743000	0.098966000
O	-1.449624000	-1.630107000	0.093263000
H	-0.426607000	-1.645177000	0.015110000
C	-1.202278000	3.324373000	-0.264788000
H	-0.740470000	3.710784000	0.646916000
H	-0.483569000	3.465120000	-1.075389000
H	-2.095900000	3.912578000	-0.476335000
C	0.640913000	0.432562000	-0.037469000
N	0.983000000	-0.825873000	-0.086349000
C	2.301922000	-1.326315000	-0.028429000
C	2.641782000	-2.372223000	-0.897608000
C	3.259643000	-0.917201000	0.909844000
C	3.900826000	-2.956366000	-0.866104000
C	4.514539000	-1.512022000	0.955779000
H	3.018207000	-0.140854000	1.624058000
C	4.862651000	-2.535706000	0.063680000
H	5.235126000	-1.178692000	1.696151000
C	1.618664000	1.567935000	-0.026744000
C	1.723453000	2.409379000	1.085428000
C	2.438131000	1.797459000	-1.136888000
C	2.641485000	3.457213000	1.092395000
H	1.092260000	2.235523000	1.950246000
C	3.344028000	2.855190000	-1.135120000
H	2.365308000	1.145956000	-2.000002000
C	3.450922000	3.684738000	-0.019597000
H	2.722386000	4.096901000	1.964176000
H	3.970015000	3.028035000	-2.003232000
H	4.160888000	4.504067000	-0.017026000
H	1.902542000	-2.719573000	-1.610782000
H	4.140549000	-3.751690000	-1.564817000
N	6.148011000	-3.087591000	0.067992000
H	6.654106000	-3.003744000	0.937476000
H	6.224712000	-4.014183000	-0.325428000

Structure 5a

C	-1.247973000	2.027487000	-0.484785000
C	-0.336641000	0.911532000	-0.351897000
C	-1.202110000	-0.250175000	-0.169399000
N	-2.488669000	0.257670000	-0.199005000
N	-2.489460000	1.634410000	-0.398535000
C	-3.725416000	-0.420038000	-0.062043000
C	-3.776639000	-1.811915000	0.099367000
C	-4.915220000	0.320415000	-0.089845000
C	-5.012240000	-2.441628000	0.230844000
H	-2.860591000	-2.381893000	0.120186000
C	-6.138222000	-0.328324000	0.042994000
H	-4.865520000	1.392056000	-0.216321000
C	-6.198281000	-1.711777000	0.204556000
H	-5.040291000	-3.518738000	0.354599000
H	-7.051090000	0.256805000	0.019533000
H	-7.154323000	-2.212084000	0.307504000
O	-0.871012000	-1.441147000	-0.007643000
H	0.860753000	-1.183626000	-0.180211000
C	-0.989469000	3.482671000	-0.732103000
H	-0.409904000	3.939402000	0.074127000
H	-0.446835000	3.643156000	-1.668716000
H	-1.948405000	3.997293000	-0.800754000
C	1.053798000	0.795087000	-0.370980000
N	1.578514000	-0.444578000	-0.266792000
C	2.948631000	-0.832751000	-0.379081000
C	3.625459000	-0.741540000	-1.595731000
C	3.586045000	-1.392722000	0.747638000
C	4.944606000	-1.166590000	-1.714399000
C	4.914114000	-1.828044000	0.611338000
C	5.583148000	-1.711872000	-0.599774000
H	5.419245000	-2.253554000	1.472426000
H	3.095733000	-0.348437000	-2.456159000
H	5.459317000	-1.090034000	-2.664159000
C	1.993631000	1.972596000	-0.447297000
H	1.442160000	2.851605000	-0.772438000
H	2.751372000	1.773169000	-1.208106000
C	2.715703000	2.278219000	0.889425000
H	3.450858000	3.063195000	0.684334000
C	1.794042000	2.726979000	2.026033000
H	1.271290000	3.655205000	1.777089000
H	1.038602000	1.972477000	2.260026000
H	2.372007000	2.909139000	2.935686000
H	3.285381000	1.400404000	1.203136000
H	6.608331000	-2.056376000	-0.675459000
N	2.937740000	-1.450173000	1.975052000
H	1.929780000	-1.399092000	1.957564000
H	3.291539000	-2.127846000	2.633035000

Structure 5b

C	-1.240762000	2.053101000	-0.525109000
C	-0.335555000	0.948932000	-0.369617000
C	-1.189986000	-0.153522000	-0.183549000
N	-2.468826000	0.289029000	-0.221942000
N	-2.494326000	1.658138000	-0.433040000
C	-3.692420000	-0.418229000	-0.057592000
C	-3.739361000	-1.814744000	-0.129861000
C	-4.863918000	0.312693000	0.167651000
C	-4.959619000	-2.466984000	0.032742000
H	-2.839040000	-2.382874000	-0.307782000
C	-6.073371000	-0.355956000	0.322991000
H	-4.810727000	1.390981000	0.210965000
C	-6.130263000	-1.747577000	0.259341000
H	-4.988493000	-3.549292000	-0.023936000
H	-6.976456000	0.218232000	0.496992000
H	-7.075252000	-2.263713000	0.382761000
O	-0.830970000	-1.407945000	0.001715000
H	0.195656000	-1.348439000	-0.052343000
C	-0.982941000	3.505064000	-0.789387000
H	-0.385056000	3.963676000	0.001465000
H	-0.457199000	3.654865000	-1.736821000
H	-1.939916000	4.024843000	-0.844774000
C	1.100697000	0.772636000	-0.389237000
N	1.525399000	-0.451547000	-0.214948000
C	2.881076000	-0.848977000	-0.321044000
C	3.546430000	-0.870760000	-1.549903000
C	3.525095000	-1.363943000	0.825802000
C	4.844273000	-1.364347000	-1.658756000
C	4.830718000	-1.856651000	0.704519000
C	5.485380000	-1.855884000	-0.522657000
H	5.332197000	-2.240846000	1.587432000
H	3.021085000	-0.517820000	-2.430666000
H	5.341391000	-1.376201000	-2.621401000
C	2.037119000	1.953135000	-0.566412000
H	1.481051000	2.812702000	-0.938284000
H	2.777009000	1.699338000	-1.327828000
C	2.789509000	2.338706000	0.730149000
H	3.524783000	3.105477000	0.464469000
C	1.897128000	2.860207000	1.859559000
H	1.374772000	3.776825000	1.569281000
H	1.143545000	2.125392000	2.155299000
H	2.496101000	3.090287000	2.744520000
H	3.359446000	1.476017000	1.082725000
H	6.494059000	-2.247272000	-0.590613000
N	2.884495000	-1.307957000	2.064832000
H	1.877179000	-1.239910000	2.017000000
H	3.199058000	-1.983741000	2.745719000

Structure 6a

C	-1.641581000	2.098143000	-0.309225000
C	-0.652996000	1.041647000	-0.303490000
C	-1.432109000	-0.190003000	-0.209236000
N	-2.751481000	0.227601000	-0.163479000
N	-2.850952000	1.612970000	-0.232456000
C	-3.934526000	-0.544825000	-0.063985000
C	-3.886103000	-1.945764000	-0.026664000
C	-5.172997000	0.109004000	-0.002650000
C	-5.071794000	-2.670307000	0.071560000
H	-2.932656000	-2.448923000	-0.074032000
C	-6.344772000	-0.633723000	0.094979000
H	-5.200252000	1.188450000	-0.033544000
C	-6.305608000	-2.026824000	0.133271000
H	-5.022511000	-3.753423000	0.099257000
H	-7.295999000	-0.114743000	0.141402000
H	-7.222091000	-2.600614000	0.209387000
O	-1.018229000	-1.363954000	-0.164571000
H	0.692111000	-0.966137000	-0.288294000
C	-1.492582000	3.585441000	-0.420423000
H	-0.920925000	4.004688000	0.411725000
H	-0.993181000	3.874076000	-1.350337000
H	-2.486932000	4.033108000	-0.414062000
C	0.742099000	1.026103000	-0.367748000
N	1.352294000	-0.175764000	-0.368462000
C	2.734933000	-0.474587000	-0.568502000
C	3.423151000	-1.200228000	0.417157000
C	3.352232000	-0.109668000	-1.768798000
C	4.768977000	-1.551527000	0.169495000
C	4.684915000	-0.443680000	-1.981505000
C	5.390695000	-1.158051000	-1.022793000
C	1.587176000	2.277532000	-0.364040000
H	0.952019000	3.137098000	-0.562409000
H	2.311903000	2.225106000	-1.178335000
C	2.360073000	2.501066000	0.959221000
H	2.998255000	3.378838000	0.814262000
C	1.473941000	2.712578000	2.189458000
H	0.838600000	3.596220000	2.078739000
H	0.819640000	1.856426000	2.373121000
H	2.087353000	2.859390000	3.082346000
H	3.036990000	1.659656000	1.128239000
H	6.427567000	-1.426822000	-1.197118000
C	2.780663000	-1.602177000	1.720484000
H	1.797070000	-1.152444000	1.842640000
H	2.647090000	-2.689360000	1.787479000
H	3.400655000	-1.297047000	2.570224000
N	5.499872000	-2.233856000	1.144223000
H	4.977033000	-2.836752000	1.761250000
H	6.353523000	-2.664582000	0.821397000
H	2.779864000	0.398020000	-2.534566000
H	5.169864000	-0.166142000	-2.910382000

Structure 6b

C	-1.626119000	2.112755000	-0.377561000
C	-0.650308000	1.060415000	-0.346826000
C	-1.427009000	-0.106572000	-0.226884000
N	-2.732115000	0.254969000	-0.184215000
N	-2.849226000	1.632267000	-0.278932000
C	-3.903384000	-0.539853000	-0.043777000
C	-3.859997000	-1.931120000	-0.188536000
C	-5.117387000	0.098300000	0.234019000
C	-5.032332000	-2.670337000	-0.046651000
H	-2.927062000	-2.428347000	-0.405726000
C	-6.277647000	-0.656451000	0.368298000
H	-5.134576000	1.173869000	0.334495000
C	-6.244358000	-2.043551000	0.231454000
H	-4.990448000	-3.747838000	-0.159629000
H	-7.213616000	-0.153128000	0.583366000
H	-7.151363000	-2.627113000	0.338564000
O	-0.982875000	-1.344259000	-0.159227000
H	0.038433000	-1.210057000	-0.236187000
C	-1.468014000	3.595330000	-0.526383000
H	-0.874538000	4.026296000	0.283420000
H	-0.983674000	3.856066000	-1.471891000
H	-2.456784000	4.054920000	-0.511619000
C	0.794000000	0.977118000	-0.421085000
N	1.292705000	-0.227875000	-0.379135000
C	2.663525000	-0.540689000	-0.553878000
C	3.380782000	-1.133984000	0.500941000
C	3.257207000	-0.365419000	-1.811999000
C	4.717948000	-1.522493000	0.273946000
C	4.576159000	-0.755481000	-2.014216000
C	5.308629000	-1.325777000	-0.981454000
C	1.648113000	2.229941000	-0.498607000
H	1.025431000	3.081906000	-0.768381000
H	2.379147000	2.103212000	-1.299300000
C	2.414188000	2.539935000	0.809075000
H	3.060451000	3.401819000	0.612176000
C	1.526619000	2.836913000	2.020928000
H	0.900125000	3.718142000	1.853864000
H	0.864417000	1.999372000	2.256216000
H	2.138157000	3.033562000	2.905634000
H	3.082663000	1.705309000	1.034777000
H	6.338404000	-1.628367000	-1.141832000
C	2.763519000	-1.351037000	1.859574000
H	1.739724000	-0.983382000	1.893686000
H	2.734238000	-2.416104000	2.124271000
H	3.334170000	-0.839591000	2.643244000
N	5.476865000	-2.051445000	1.323795000
H	4.965476000	-2.540244000	2.043222000
H	6.306508000	-2.552350000	1.041501000
H	2.671349000	0.046889000	-2.624472000
H	5.033956000	-0.623846000	-2.988429000

Structure 7a

C	-1.527541000	1.969067000	-0.531831000
C	-0.567462000	0.910797000	-0.305529000
C	-1.380535000	-0.280655000	-0.076524000
N	-2.690277000	0.160070000	-0.175845000
N	-2.751135000	1.520111000	-0.458230000
C	-3.895610000	-0.570081000	-0.036177000
C	-3.884141000	-1.947488000	0.226893000
C	-5.119240000	0.102009000	-0.163842000
C	-5.091107000	-2.630466000	0.358155000
H	-2.942157000	-2.464658000	0.325447000
C	-6.312716000	-0.599220000	-0.029277000
H	-5.117979000	1.162951000	-0.367085000
C	-6.310284000	-1.968608000	0.232517000
H	-5.070159000	-3.695631000	0.561495000
H	-7.251972000	-0.066377000	-0.130551000
H	-7.243544000	-2.510093000	0.336523000
O	-0.999884000	-1.440919000	0.165889000
H	0.732324000	-1.092842000	0.099375000
C	-1.335812000	3.420045000	-0.855478000
H	-0.803780000	3.951646000	-0.062127000
H	-0.776014000	3.557253000	-1.785748000
H	-2.317597000	3.878738000	-0.977569000
C	0.828110000	0.864589000	-0.279519000
N	1.410145000	-0.331555000	-0.064498000
C	2.795674000	-0.666713000	-0.108401000
C	3.550101000	-0.526669000	-1.275945000
C	3.400423000	-1.235479000	1.015686000
C	4.885857000	-0.907740000	-1.308213000
C	4.731135000	-1.631842000	0.983852000
H	2.819134000	-1.366078000	1.921528000
C	5.500984000	-1.466883000	-0.177778000
H	5.181862000	-2.067183000	1.869673000
H	3.083512000	-0.139147000	-2.174054000
H	5.454905000	-0.786802000	-2.224236000
N	6.853342000	-1.803642000	-0.192870000
H	7.150085000	-2.480585000	0.494095000
H	7.259338000	-1.971537000	-1.101294000
C	1.701980000	2.088728000	-0.414966000
H	1.101971000	2.915566000	-0.786490000
H	2.474726000	1.901013000	-1.163155000
C	2.395399000	2.506574000	0.905500000
H	3.047044000	3.354902000	0.671618000
C	1.438659000	2.893452000	2.036084000
H	0.811133000	3.745251000	1.758014000
H	0.775237000	2.068370000	2.307861000
H	1.999455000	3.177113000	2.930581000
H	3.053770000	1.700090000	1.237868000

Structure 7b

C	-1.533296000	2.004934000	-0.555374000
C	-0.570650000	0.963539000	-0.335365000
C	-1.363105000	-0.178791000	-0.116021000
N	-2.665895000	0.187705000	-0.196742000
N	-2.764301000	1.542441000	-0.471454000
C	-3.851116000	-0.581035000	-0.032251000
C	-3.806560000	-1.976702000	0.064673000
C	-5.080521000	0.085579000	0.023985000
C	-4.993097000	-2.689739000	0.221989000
H	-2.862002000	-2.496657000	0.020517000
C	-6.254111000	-0.644141000	0.178699000
H	-5.098071000	1.162590000	-0.058366000
C	-6.220284000	-2.034251000	0.280235000
H	-4.949579000	-3.770517000	0.296934000
H	-7.201388000	-0.118258000	0.221253000
H	-7.137947000	-2.597971000	0.401928000
O	-0.931554000	-1.396190000	0.131781000
H	0.098462000	-1.267241000	0.098747000
C	-1.352980000	3.457417000	-0.876558000
H	-0.813710000	3.986440000	-0.086931000
H	-0.801852000	3.598537000	-1.810870000
H	-2.336476000	3.915094000	-0.987908000
C	0.873836000	0.868759000	-0.307292000
N	1.354469000	-0.327078000	-0.098101000
C	2.731045000	-0.657758000	-0.111859000
C	3.508245000	-0.573393000	-1.273510000
C	3.320367000	-1.205851000	1.033891000
C	4.836460000	-0.986152000	-1.279438000
C	4.649514000	-1.611287000	1.034056000
H	2.724289000	-1.308676000	1.933817000
C	5.433751000	-1.506081000	-0.123391000
H	5.084423000	-2.018262000	1.941561000
H	3.061191000	-0.205706000	-2.190273000
H	5.415097000	-0.910680000	-2.194868000
N	6.787739000	-1.863448000	-0.110224000
H	7.050500000	-2.533354000	0.598092000
H	7.181536000	-2.097279000	-1.010083000
C	1.741013000	2.106188000	-0.457059000
H	1.142044000	2.926410000	-0.850353000
H	2.522515000	1.906288000	-1.192168000
C	2.418742000	2.545824000	0.862564000
H	3.068840000	3.395512000	0.628330000
C	1.451085000	2.938356000	1.982328000
H	0.819734000	3.783255000	1.691310000
H	0.791896000	2.110956000	2.258235000
H	2.002355000	3.234893000	2.878715000
H	3.076452000	1.744317000	1.207973000

Structure 8a

C	5.994102000	-0.243492000	-0.511781000
C	4.624863000	-0.170698000	-0.045654000
C	4.379226000	1.257075000	0.166124000
N	5.581104000	1.868044000	-0.138316000
N	6.534126000	0.942373000	-0.557085000
C	5.921205000	3.244050000	-0.109284000
C	4.997622000	4.209279000	0.315720000
C	7.205083000	3.637563000	-0.509955000
C	5.369534000	5.551537000	0.333357000
H	4.008262000	3.906710000	0.622602000
C	7.555719000	4.983248000	-0.484330000
H	7.909709000	2.885975000	-0.834743000
C	6.643664000	5.950091000	-0.063911000
H	4.647460000	6.290314000	0.663253000
H	8.552413000	5.274807000	-0.796991000
H	6.922311000	6.997422000	-0.046383000
O	3.322421000	1.806803000	0.521786000
H	2.335074000	0.338787000	0.517474000
C	6.809917000	-1.420570000	-0.947812000
H	7.039132000	-2.088358000	-0.113840000
H	6.287685000	-2.012904000	-1.703207000
H	7.747849000	-1.056657000	-1.368438000
C	3.628356000	-1.123205000	0.130867000
N	2.389367000	-0.691788000	0.462511000
C	1.216557000	-1.402994000	0.805689000
C	-0.000165000	-0.806990000	0.458878000
C	1.215210000	-2.610657000	1.512569000
C	-1.216913000	-1.402859000	0.805920000
C	-0.000157000	-3.198740000	1.847928000
C	-1.215532000	-2.610523000	1.512811000
H	-0.000156000	0.126874000	-0.092451000
H	-2.141903000	-3.072470000	1.819711000
H	-0.000160000	-4.124519000	2.411735000
H	2.141624000	-3.072673000	1.819258000
N	-2.389613000	-0.691465000	0.462843000
C	-3.629027000	-1.122418000	0.132180000
C	-5.994500000	-0.241852000	-0.510249000
C	-4.378335000	1.258412000	0.165256000
C	-4.624987000	-0.169437000	-0.044876000
N	-6.533782000	0.944311000	-0.556592000
N	-5.579955000	1.869828000	-0.139302000
C	-5.919152000	3.246083000	-0.111613000
C	-7.202972000	3.639991000	-0.512083000
C	-4.994729000	4.211176000	0.311868000
C	-7.552717000	4.985932000	-0.487771000
H	-7.908248000	2.888505000	-0.835693000
C	-5.365756000	5.553696000	0.328208000
H	-4.005415000	3.908304000	0.618599000
C	-6.639823000	5.952642000	-0.068869000
H	-8.549374000	5.277797000	-0.800259000

H	-4.643037000	6.292365000	0.656932000
H	-6.917778000	7.000173000	-0.052360000
O	-3.321023000	1.807846000	0.519884000
H	-2.334683000	0.339136000	0.516689000
C	-6.811319000	-1.418879000	-0.944538000
H	-6.289947000	-2.012312000	-1.699665000
H	-7.040399000	-2.085669000	-0.109731000
H	-7.749298000	-1.054817000	-1.364929000
C	3.872311000	-2.577228000	-0.089531000
C	4.694046000	-3.300922000	0.780037000
C	3.280801000	-3.226994000	-1.178210000
C	4.915904000	-4.659607000	0.567066000
H	5.149242000	-2.800314000	1.627136000
C	3.516410000	-4.581637000	-1.397084000
H	2.643836000	-2.669315000	-1.855203000
C	4.330540000	-5.301310000	-0.523174000
H	5.548412000	-5.214953000	1.250206000
H	3.063488000	-5.075146000	-2.249341000
H	4.509125000	-6.357067000	-0.692199000
C	-3.874121000	-2.576490000	-0.086642000
C	-3.283978000	-3.227677000	-1.175212000
C	-4.695599000	-3.298806000	0.784312000
C	-3.520660000	-4.582376000	-1.392585000
H	-2.647223000	-2.671066000	-1.853282000
C	-4.918540000	-4.657546000	0.572823000
H	-5.149747000	-2.797085000	1.631316000
C	-4.334520000	-5.300678000	-0.517296000
H	-3.068785000	-5.076999000	-2.244753000
H	-5.550843000	-5.211818000	1.257023000
H	-4.513944000	-6.356478000	-0.685159000

Structure 8b

C	5.853456000	-0.405028000	-0.781765000
C	4.594392000	-0.189645000	-0.127412000
C	4.601912000	1.184687000	0.169420000
N	5.780036000	1.699706000	-0.252396000
N	6.548783000	0.711776000	-0.850227000
C	6.268268000	3.035088000	-0.203032000
C	5.674509000	3.993597000	0.625249000
C	7.371668000	3.375767000	-0.992673000
C	6.186232000	5.289081000	0.647415000
H	4.825045000	3.735013000	1.239117000
C	7.871538000	4.672934000	-0.953327000
H	7.823513000	2.620021000	-1.618834000
C	7.282755000	5.637808000	-0.137094000
H	5.720157000	6.027319000	1.290180000
H	8.726864000	4.928908000	-1.568487000
H	7.675071000	6.647804000	-0.111945000
O	3.632648000	1.880015000	0.731641000
H	2.890933000	1.187609000	0.833452000

C	6.435654000	-1.649620000	-1.376074000
H	6.696257000	-2.381307000	-0.607685000
H	5.732536000	-2.133586000	-2.057851000
H	7.340202000	-1.387449000	-1.925822000
C	3.445775000	-1.008495000	0.184139000
N	2.401589000	-0.387699000	0.659821000
C	1.218408000	-1.010304000	1.112146000
C	0.000000000	-0.489504000	0.663088000
C	1.212487000	-2.032935000	2.071384000
C	-1.218417000	-1.010320000	1.112123000
C	-0.000004000	-2.526083000	2.544000000
C	-1.212491000	-2.032952000	2.071362000
H	-0.000004000	0.329070000	-0.047302000
H	-2.146438000	-2.414878000	2.461883000
H	-0.000007000	-3.302305000	3.301409000
H	2.146441000	-2.414830000	2.461920000
N	-2.401576000	-0.387722000	0.659753000
C	-3.445841000	-1.008492000	0.184222000
C	-5.853413000	-0.404842000	-0.781849000
C	-4.601847000	1.184768000	0.169464000
C	-4.594409000	-0.189565000	-0.127343000
N	-6.548690000	0.711997000	-0.850289000
N	-5.779921000	1.699865000	-0.252393000
C	-6.268064000	3.035287000	-0.203086000
C	-7.371202000	3.376088000	-0.993037000
C	-5.674509000	3.993691000	0.625466000
C	-7.871004000	4.673284000	-0.953746000
H	-7.822902000	2.620416000	-1.619390000
C	-6.186160000	5.289205000	0.647568000
H	-4.825258000	3.735007000	1.239583000
C	-7.282415000	5.638059000	-0.137259000
H	-8.726126000	4.929359000	-1.569148000
H	-5.720244000	6.027365000	1.290537000
H	-7.674675000	6.648078000	-0.112155000
O	-3.632540000	1.880066000	0.731684000
H	-2.890795000	1.187735000	0.833371000
C	-6.435625000	-1.649348000	-1.376325000
H	-5.732655000	-2.132995000	-2.058488000
H	-6.695860000	-2.381314000	-0.608084000
H	-7.340386000	-1.387135000	-1.925702000
C	3.492091000	-2.484102000	-0.072358000
C	4.348623000	-3.308104000	0.664484000
C	2.675882000	-3.051032000	-1.056259000
C	4.379472000	-4.680650000	0.429566000
H	4.983229000	-2.874406000	1.429741000
C	2.720923000	-4.421186000	-1.302249000
H	2.008897000	-2.417079000	-1.628952000
C	3.569036000	-5.239400000	-0.557621000
H	5.038005000	-5.312657000	1.014777000
H	2.090987000	-4.850252000	-2.073256000
H	3.598650000	-6.306680000	-0.745545000

C	-3.492306000	-2.484111000	-0.072163000
C	-2.676077000	-3.051238000	-1.055935000
C	-4.348976000	-3.307946000	0.664709000
C	-2.721247000	-4.421410000	-1.301784000
H	-2.008980000	-2.417414000	-1.628641000
C	-4.379945000	-4.680517000	0.429945000
H	-4.983577000	-2.874098000	1.429884000
C	-3.569498000	-5.239457000	-0.557126000
H	-2.091302000	-4.850631000	-2.072697000
H	-5.038579000	-5.312391000	1.015184000
H	-3.599204000	-6.306753000	-0.744938000

Structure 9a

C	-4.995342000	0.832399000	-1.684800000
C	-4.169102000	0.478654000	-0.548819000
C	-4.895726000	-0.608516000	0.108485000
N	-6.032658000	-0.788542000	-0.657289000
N	-6.064144000	0.087042000	-1.737262000
C	-7.098660000	-1.707134000	-0.481797000
C	-7.094293000	-2.629187000	0.574066000
C	-8.170944000	-1.686760000	-1.383747000
C	-8.159593000	-3.515930000	0.713016000
H	-6.268728000	-2.644856000	1.268751000
C	-9.224535000	-2.580958000	-1.226582000
H	-8.164580000	-0.973332000	-2.194815000
C	-9.228770000	-3.501224000	-0.179452000
H	-8.146315000	-4.225671000	1.532863000
1	-10.048179000	-2.555054000	-1.931594000
1	-10.052430000	-4.195919000	-0.061980000
O	-4.579790000	-1.230067000	1.139269000
H	-3.088300000	-0.371762000	1.433785000
C	-4.794272000	1.845863000	-2.770387000
H	-4.755122000	2.865826000	-2.378391000
H	-3.871036000	1.663568000	-3.328880000
H	-5.631904000	1.778048000	-3.465171000
C	-2.959636000	0.957301000	-0.052235000
N	-2.458867000	0.354678000	1.050105000
C	-1.211603000	0.541854000	1.700921000
C	0.000016000	0.419336000	1.014065000
C	-1.208652000	0.731600000	3.086921000
C	1.211655000	0.541843000	1.700887000
C	0.000058000	0.813140000	3.771104000
C	1.208748000	0.731591000	3.086887000
H	0.000002000	0.152942000	-0.034395000
C	-2.245524000	2.156853000	-0.626173000
H	-2.683350000	2.402754000	-1.590089000
H	-1.200341000	1.905395000	-0.815943000
C	-2.293331000	3.401877000	0.293978000
H	-1.688632000	4.179610000	-0.184048000
C	-3.700676000	3.941720000	0.560519000

H	-4.197692000	4.241215000	-0.366714000
H	-4.334759000	3.199412000	1.051775000
H	-3.656024000	4.820865000	1.208382000
H	-1.800642000	3.172401000	1.242582000
H	2.151788000	0.804456000	3.615468000
H	0.000074000	0.950945000	4.845973000
H	-2.151677000	0.804471000	3.615530000
N	2.458897000	0.354660000	1.050028000
C	2.959618000	0.957271000	-0.052339000
C	4.995193000	0.832194000	-1.685068000
C	4.895798000	-0.608395000	0.108494000
C	4.169060000	0.478608000	-0.548957000
N	6.064027000	0.086879000	-1.737479000
N	6.032674000	-0.788508000	-0.657343000
C	7.098727000	-1.707028000	-0.481783000
C	8.170864000	-1.686864000	-1.383912000
C	7.094560000	-2.628799000	0.574328000
C	9.224507000	-2.580989000	-1.226680000
H	8.164349000	-0.973650000	-2.195168000
C	8.159908000	-3.515474000	0.713341000
H	6.269107000	-2.644309000	1.269149000
C	9.228939000	-3.500975000	-0.179305000
H	10.048036000	-2.555248000	-1.931833000
H	8.146784000	-4.224996000	1.533379000
H	10.052639000	-4.195615000	-0.061783000
O	4.579993000	-1.229735000	1.139444000
H	3.088326000	-0.371805000	1.433668000
C	4.793992000	1.845447000	-2.770828000
H	3.870729000	1.662998000	-3.329226000
H	4.754813000	2.865480000	-2.379019000
H	5.631580000	1.777549000	-3.465656000
C	2.245442000	2.156780000	-0.626289000
H	1.200276000	1.905255000	-0.816061000
H	2.683256000	2.402707000	-1.590203000
C	2.293151000	3.401805000	0.293869000
H	1.688411000	4.179500000	-0.184165000
H	1.800454000	3.172290000	1.242459000
C	3.700452000	3.941742000	0.560453000
H	4.197469000	4.241289000	-0.366763000
H	3.655723000	4.820872000	1.208330000
H	4.334576000	3.199470000	1.051711000

Structure 9b

C	5.074326000	-0.790884000	-1.682396000
C	4.189098000	-0.525926000	-0.583373000
C	4.774344000	0.585723000	0.050952000
N	5.899665000	0.915842000	-0.625632000
N	6.079597000	0.060446000	-1.700267000

C	6.857774000	1.938048000	-0.375205000
C	6.585113000	2.988997000	0.507217000
C	8.086451000	1.882085000	-1.041928000
C	7.550100000	3.971234000	0.719046000
H	5.637804000	3.038453000	1.022049000
C	9.035602000	2.874108000	-0.820613000
H	8.276745000	1.068297000	-1.726688000
C	8.776438000	3.922586000	0.061043000
H	7.332261000	4.782310000	1.404670000
H	9.985111000	2.823118000	-1.341533000
H	9.520301000	4.692228000	0.231160000
O	4.331980000	1.208805000	1.123798000
H	3.472140000	0.693521000	1.339151000
C	5.008078000	-1.816221000	-2.773112000
H	5.037338000	-2.835186000	-2.379699000
H	4.096446000	-1.712604000	-3.368797000
H	5.864365000	-1.677898000	-3.433903000
C	2.965532000	-1.106234000	-0.073038000
N	2.445211000	-0.511066000	0.964501000
C	1.217841000	-0.858453000	1.570497000
C	-0.000124000	-0.614813000	0.922642000
C	1.210934000	-1.328208000	2.890262000
C	-1.218193000	-0.858492000	1.570272000
C	-0.000346000	-1.565085000	3.533875000
C	-1.211519000	-1.328276000	2.890035000
H	-0.000039000	-0.181686000	-0.070984000
C	2.376564000	-2.359902000	-0.694103000
H	2.811837000	-2.521071000	-1.679354000
H	1.305800000	-2.204783000	-0.842589000
C	2.567547000	-3.627746000	0.172404000
H	2.048498000	-4.449504000	-0.332584000
C	4.025026000	-4.024668000	0.422201000
H	4.549508000	-4.241833000	-0.512904000
H	4.578444000	-3.235666000	0.938136000
H	4.075577000	-4.923572000	1.042317000
H	2.057048000	-3.484673000	1.128076000
H	-2.153756000	-1.488119000	3.400616000
H	-0.000424000	-1.925632000	4.556645000
H	2.153084000	-1.487967000	3.401037000
N	-2.445466000	-0.511186000	0.964049000
C	-2.965328000	-1.105953000	-0.073962000
C	-5.073974000	-0.790533000	-1.683532000
C	-4.774395000	0.585683000	0.050220000
C	-4.188925000	-0.525751000	-0.584304000
N	-6.079352000	0.060662000	-1.701311000
N	-5.899684000	0.915794000	-0.626416000
C	-6.858023000	1.937737000	-0.375805000
C	-8.086855000	1.881386000	-1.042208000
C	-6.585442000	2.988798000	0.506495000
C	-9.036232000	2.873151000	-0.820702000
H	-8.277088000	1.067519000	-1.726892000

C	-7.550650000	3.970763000	0.718540000
H	-5.638008000	3.038558000	1.021082000
C	-8.777145000	3.921738000	0.060849000
H	-9.985855000	2.821861000	-1.341384000
H	-7.332872000	4.781931000	1.404076000
H	-9.521181000	4.691175000	0.231134000
O	-4.332157000	1.208580000	1.123203000
H	-3.472117000	0.693354000	1.338395000
C	-5.007463000	-1.815572000	-2.774513000
H	-4.096007000	-1.711408000	-3.370376000
H	-5.036153000	-2.834631000	-2.381303000
H	-5.863960000	-1.677514000	-3.435087000
C	-2.375854000	-2.359195000	-0.695418000
H	-1.305103000	-2.203705000	-0.843636000
H	-2.810868000	-2.520120000	-1.680811000
C	-2.566664000	-3.627419000	0.170567000
H	-2.047367000	-4.448861000	-0.334676000
H	-2.056322000	-3.484621000	1.126367000
C	-4.024108000	-4.024739000	0.419974000
H	-4.548425000	-4.241536000	-0.515312000
H	-4.074588000	-4.923973000	1.039618000
H	-4.577730000	-3.236099000	0.936230000

Atomic coordinates for the imine-one(I) and imine-one(II) tautomeric forms presented in Figure S1

Structure S1a

C	-0.751721000	2.346527000	-0.159081000
C	-1.032701000	0.414924000	1.150480000
N	-2.021045000	0.600632000	0.195128000
N	-1.810815000	1.753963000	-0.570684000
C	-3.140029000	-0.220602000	-0.104194000
C	-3.437226000	-1.345659000	0.677510000
C	-3.957908000	0.106517000	-1.193820000
C	-4.545293000	-2.126895000	0.358533000
H	-2.808440000	-1.600704000	1.516302000
C	-5.059918000	-0.687380000	-1.494156000
H	-3.724341000	0.975117000	-1.791778000
C	-5.362648000	-1.808081000	-0.723071000
H	-4.766658000	-2.994950000	0.969478000
H	-5.684486000	-0.423819000	-2.340468000
H	-6.222351000	-2.423173000	-0.962150000
O	-0.933073000	-0.491660000	1.949017000
C	-0.269230000	3.611632000	-0.784273000
H	-0.200533000	4.414698000	-0.043021000
H	0.728033000	3.478504000	-1.215811000
H	-0.953318000	3.918731000	-1.575545000
C	1.358872000	1.231168000	0.767622000
C	2.400845000	2.130562000	1.385299000
H	2.372614000	3.131230000	0.940571000
H	2.186638000	2.251515000	2.451950000
H	3.404064000	1.725333000	1.265269000
N	1.551672000	0.164442000	0.102872000
C	2.818175000	-0.344270000	-0.243182000
C	3.080872000	-1.685354000	0.127815000
C	3.738354000	0.352643000	-1.030332000
C	4.281126000	-2.273105000	-0.294447000
C	4.921295000	-0.249760000	-1.453551000
H	3.499783000	1.363263000	-1.343647000
C	5.187921000	-1.565834000	-1.077209000
H	4.496670000	-3.296338000	-0.003415000
H	5.618296000	0.298389000	-2.076002000
H	6.103834000	-2.048640000	-1.398970000
N	2.186455000	-2.352118000	0.950080000
H	1.259678000	-1.949186000	1.012366000
H	2.199682000	-3.359520000	0.908246000
C	-0.099399000	1.624651000	0.997820000
H	-0.176774000	2.234286000	1.906128000

Structure S1b

C	-0.940331000	2.226351000	-0.175146000
C	-0.110315000	1.155581000	-0.023550000
C	-0.939885000	-0.065593000	-0.077852000
N	-2.254936000	0.408134000	-0.290951000
C	-3.470285000	-0.295946000	-0.125462000
C	-3.538787000	-1.652772000	-0.469414000
C	-4.610978000	0.362485000	0.350615000
C	-4.743467000	-2.333377000	-0.326901000
H	-2.653518000	-2.158313000	-0.825096000
C	-5.811716000	-0.332736000	0.472959000
H	-4.553064000	1.402669000	0.644010000
C	-5.885903000	-1.682223000	0.137756000
H	-4.787457000	-3.384351000	-0.589450000
H	-6.688390000	0.185918000	0.844445000
H	-6.820697000	-2.220924000	0.239000000
O	-0.654114000	-1.239372000	0.075527000
C	-0.668970000	3.692773000	-0.276739000
H	-0.686862000	4.164902000	0.710289000
H	0.305187000	3.881401000	-0.725988000
H	-1.428053000	4.188237000	-0.888518000
C	1.345345000	1.117846000	0.204073000
C	1.974854000	2.201054000	1.059091000
H	2.247480000	3.080908000	0.467257000
H	1.281416000	2.527485000	1.836992000
H	2.887708000	1.828644000	1.526150000
N	1.949854000	0.102611000	-0.285526000
C	3.304340000	-0.207915000	-0.154795000
C	3.572711000	-1.572050000	0.146753000
C	4.367203000	0.662182000	-0.422487000
C	4.907028000	-2.000743000	0.189904000
C	5.686790000	0.215168000	-0.393478000
H	4.150257000	1.686661000	-0.702511000
C	5.948881000	-1.118996000	-0.080050000
H	5.120812000	-3.037248000	0.431488000
H	6.497518000	0.897075000	-0.621209000
H	6.971441000	-1.479215000	-0.052394000
N	2.516087000	-2.407825000	0.445905000
H	1.591886000	-2.067781000	0.195237000
H	2.663622000	-3.397489000	0.327476000
H	-2.827073000	2.217792000	-0.986977000
N	-2.262204000	1.807374000	-0.247440000

Structure S2a

C	-1.003687000	-1.007810000	1.692461000
C	-1.455502000	-1.001446000	-0.614570000
N	-2.559838000	-0.731732000	0.181436000
N	-2.249181000	-0.738799000	1.548978000
C	-3.891942000	-0.436196000	-0.212574000

C	-4.275568000	-0.496475000	-1.559702000
C	-4.831850000	-0.083647000	0.765124000
C	-5.591192000	-0.201787000	-1.909709000
H	-3.554214000	-0.767365000	-2.314983000
C	-6.140525000	0.205702000	0.393132000
H	-4.530342000	-0.042590000	1.801458000
C	-6.530644000	0.150057000	-0.943723000
H	-5.878216000	-0.251780000	-2.954124000
H	-6.858586000	0.476779000	1.159089000
H	-7.551813000	0.376451000	-1.227508000
O	-1.404783000	-1.040249000	-1.825548000
C	-0.369622000	-1.103313000	3.038037000
H	0.071650000	-2.093916000	3.188378000
H	0.436501000	-0.370870000	3.140529000
H	-1.113204000	-0.922916000	3.814541000
C	0.981209000	-0.501793000	0.109370000
N	2.001811000	-1.214972000	-0.182463000
C	3.257359000	-0.697006000	-0.558352000
C	4.392242000	-1.327635000	0.003254000
C	3.428457000	0.282592000	-1.542875000
C	5.666859000	-0.918857000	-0.407589000
C	4.700138000	0.663831000	-1.957153000
H	2.551792000	0.722903000	-2.001563000
C	5.819800000	0.065868000	-1.377437000
H	6.539930000	-1.388119000	0.034975000
H	4.815445000	1.414477000	-2.729795000
H	6.816258000	0.357573000	-1.689665000
N	4.216609000	-2.289223000	0.998108000
H	3.305610000	-2.730925000	0.968063000
H	4.972982000	-2.952277000	1.089751000
C	0.957576000	0.985338000	0.300337000
C	1.853912000	1.568872000	1.207562000
C	0.038740000	1.805433000	-0.366825000
C	1.825642000	2.938682000	1.447340000
H	2.575555000	0.945486000	1.722687000
C	0.023596000	3.179860000	-0.135037000
H	-0.643629000	1.382510000	-1.093399000
C	0.911452000	3.749101000	0.774545000
H	2.520770000	3.374012000	2.156286000
H	-0.684319000	3.803848000	-0.668582000
H	0.893171000	4.817452000	0.957742000
C	-0.309253000	-1.265580000	0.376995000
H	-0.056070000	-2.328402000	0.292428000

Structure S2b

C	-1.133204000	1.566261000	0.846156000
C	-0.524457000	0.549133000	0.169871000
C	-1.577698000	-0.275098000	-0.452035000
N	-2.789671000	0.368874000	-0.089157000
C	-4.100014000	-0.158726000	-0.145765000

C	-4.461583000	-1.006023000	-1.202583000
C	-5.046290000	0.184085000	0.828595000
C	-5.759576000	-1.501846000	-1.266838000
H	-3.724219000	-1.278631000	-1.942735000
C	-6.345012000	-0.311405000	0.738534000
H	-4.762911000	0.810974000	1.664156000
C	-6.709850000	-1.156611000	-0.306101000
H	-6.029409000	-2.161976000	-2.083480000
H	-7.068458000	-0.041378000	1.499696000
H	-7.719931000	-1.543857000	-0.370025000
O	-1.511506000	-1.272225000	-1.141830000
C	-0.585339000	2.735404000	1.598616000
H	0.402014000	2.513831000	2.000158000
H	-0.481737000	3.608145000	0.945501000
H	-1.247395000	3.000062000	2.426426000
C	0.918345000	0.243797000	0.072777000
N	1.261168000	-0.986443000	0.194635000
C	2.548830000	-1.497946000	-0.023489000
C	2.996320000	-2.475406000	0.899134000
C	3.325455000	-1.216033000	-1.154015000
C	4.231556000	-3.097989000	0.684543000
C	4.541157000	-1.858310000	-1.366394000
H	2.951180000	-0.507498000	-1.882726000
C	4.997254000	-2.793292000	-0.436290000
H	4.582798000	-3.834602000	1.400527000
H	5.121822000	-1.638133000	-2.254445000
H	5.944621000	-3.297875000	-0.589922000
N	2.223202000	-2.742171000	2.027999000
H	1.248352000	-2.492912000	1.902098000
H	2.347190000	-3.664063000	2.421647000
C	1.848735000	1.401537000	-0.147111000
C	2.982037000	1.568767000	0.659994000
C	1.579846000	2.351624000	-1.140275000
C	3.823699000	2.661679000	0.477359000
H	3.200577000	0.839849000	1.431390000
C	2.435109000	3.434050000	-1.336993000
H	0.704872000	2.232414000	-1.769955000
C	3.556803000	3.594569000	-0.525272000
H	4.692717000	2.782534000	1.114316000
H	2.224712000	4.151628000	-2.122376000
H	4.218945000	4.440438000	-0.672055000
H	-3.065785000	2.244546000	0.630724000
N	-2.509078000	1.411309000	0.802412000

Structure S3a

C	-1.244292000	-1.173598000	1.574901000
C	-1.721339000	-0.933992000	-0.713780000
N	-2.821989000	-0.770291000	0.116244000
N	-2.495992000	-0.914243000	1.472661000
C	-4.162981000	-0.458958000	-0.232142000

C	-4.559501000	-0.379340000	-1.574601000
C	-5.099033000	-0.232637000	0.785898000
C	-5.883597000	-0.072590000	-1.879375000
H	-3.841330000	-0.553189000	-2.360854000
C	-6.416299000	0.071330000	0.458604000
H	-4.787653000	-0.299197000	1.817976000
C	-6.819113000	0.154635000	-0.873041000
H	-6.180402000	-0.013827000	-2.920605000
H	-7.131082000	0.243953000	1.255538000
H	-7.846930000	0.391765000	-1.121942000
O	-1.683829000	-0.843716000	-1.922490000
C	-0.593228000	-1.394566000	2.897526000
H	-0.124507000	-2.383099000	2.937500000
H	0.193676000	-0.654230000	3.070473000
H	-1.332477000	-1.317591000	3.695053000
C	0.718969000	-0.475798000	0.035266000
N	1.738796000	-1.141024000	-0.346801000
C	2.976807000	-0.552556000	-0.691144000
C	4.148235000	-1.052898000	-0.092901000
C	3.041043000	0.415796000	-1.702437000
C	5.389314000	-0.519795000	-0.495014000
C	4.277615000	0.908178000	-2.100630000
H	2.129588000	0.753538000	-2.178049000
C	5.445333000	0.457220000	-1.498418000
H	4.332337000	1.651284000	-2.888383000
H	6.407811000	0.854750000	-1.804144000
C	0.682628000	0.983597000	0.381896000
C	1.603123000	1.491453000	1.309728000
C	-0.271030000	1.850297000	-0.167377000
C	1.565690000	2.830486000	1.684779000
H	2.352488000	0.834715000	1.735171000
C	-0.295869000	3.194748000	0.198606000
H	-0.973529000	1.490783000	-0.908529000
C	0.616877000	3.687381000	1.128158000
H	2.281337000	3.205837000	2.407484000
H	-1.030658000	3.855953000	-0.246457000
H	0.591333000	4.732290000	1.415923000
N	6.566623000	-0.926700000	0.144795000
H	6.558934000	-1.853753000	0.543245000
H	7.415536000	-0.744846000	-0.370487000
C	4.093422000	-2.122388000	0.968200000
H	3.081021000	-2.509169000	1.074844000
H	4.740835000	-2.971951000	0.716695000
H	4.422768000	-1.745656000	1.944130000
C	-0.560876000	-1.280705000	0.233988000
H	-0.291927000	-2.324629000	0.036901000

Structure S3b

C	-1.434663000	1.360741000	1.153781000
C	-0.786336000	0.574690000	0.252629000

C	-1.799079000	-0.189080000	-0.497937000
N	-3.035929000	0.240360000	0.042426000
C	-4.305050000	-0.364392000	-0.098199000
C	-4.640792000	-1.007819000	-1.298004000
C	-5.240439000	-0.298092000	0.942730000
C	-5.901904000	-1.577848000	-1.437489000
H	-3.910834000	-1.069910000	-2.091161000
C	-6.502683000	-0.863435000	0.778028000
H	-4.974181000	0.167794000	1.882597000
C	-6.841735000	-1.506567000	-0.409523000
H	-6.151018000	-2.079370000	-2.365910000
H	-7.217316000	-0.808339000	1.591648000
H	-7.823247000	-1.949252000	-0.531959000
O	-1.679785000	-0.998083000	-1.396707000
C	-0.930131000	2.348080000	2.153337000
H	0.113307000	2.592820000	1.966005000
H	-1.510602000	3.275850000	2.118096000
H	-1.017460000	1.942494000	3.165808000
C	0.674223000	0.410394000	0.048536000
N	1.121366000	-0.787317000	0.075899000
C	2.435621000	-1.159076000	-0.263079000
C	3.172592000	-1.936291000	0.651518000
C	2.947279000	-0.873321000	-1.537585000
C	4.459475000	-2.373334000	0.281351000
C	4.208697000	-1.336557000	-1.889402000
H	2.343642000	-0.314954000	-2.241268000
C	4.970420000	-2.070655000	-0.988377000
H	4.603141000	-1.124500000	-2.877165000
H	5.960762000	-2.419068000	-1.263726000
C	1.477503000	1.659707000	-0.169442000
C	2.700059000	1.858775000	0.486415000
C	0.996009000	2.665493000	-1.018062000
C	3.420365000	3.034456000	0.297700000
H	3.086028000	1.091043000	1.145353000
C	1.728570000	3.832145000	-1.224114000
H	0.050423000	2.524600000	-1.529339000
C	2.940691000	4.022145000	-0.562552000
H	4.360736000	3.177008000	0.818128000
H	1.351808000	4.592809000	-1.898863000
H	3.507893000	4.933428000	-0.715455000
N	5.255493000	-3.068614000	1.202328000
H	4.758051000	-3.592557000	1.907079000
H	6.002617000	-3.609304000	0.791237000
C	2.609562000	-2.286838000	2.005534000
H	1.560917000	-1.999085000	2.069881000
H	2.663169000	-3.366142000	2.196361000
H	3.154062000	-1.789368000	2.817556000
N	-2.798948000	1.096274000	1.128297000
H	-3.417026000	1.903804000	1.129339000

Structure S4a

C	1.343530000	0.373226000	2.057602000
C	1.740007000	0.932904000	-0.188795000
N	2.746387000	0.182832000	0.391584000
N	2.478629000	-0.125385000	1.735902000
C	3.954027000	-0.289283000	-0.186438000
C	4.256137000	-0.041960000	-1.532984000
C	4.853973000	-1.012710000	0.607704000
C	5.451167000	-0.520132000	-2.064994000
H	3.565084000	0.514178000	-2.147020000
C	6.041437000	-1.481535000	0.055426000
H	4.613828000	-1.201885000	1.643516000
C	6.350290000	-1.239870000	-1.281882000
H	5.674944000	-0.323850000	-3.107697000
H	6.728611000	-2.040929000	0.680539000
H	7.277064000	-1.607666000	-1.706726000
O	1.687906000	1.353868000	-1.327543000
C	0.774966000	0.218836000	3.425509000
H	0.590418000	1.196539000	3.882954000
H	-0.180842000	-0.309535000	3.377257000
H	1.464140000	-0.344152000	4.055197000
C	-0.702552000	0.670396000	0.530116000
N	-0.920132000	-0.582897000	0.647358000
C	-2.079225000	-1.245458000	0.198265000
C	-2.617389000	-2.254396000	1.008123000
C	-2.648680000	-1.040915000	-1.067257000
C	-3.725794000	-2.985608000	0.603301000
C	-3.736132000	-1.793842000	-1.488346000
H	-2.222070000	-0.303025000	-1.735317000
C	-4.304781000	-2.769642000	-0.656032000
H	-4.151623000	-1.624834000	-2.476859000
C	-1.660232000	1.735598000	0.095884000
C	-1.264795000	2.731215000	-0.808000000
C	-2.949454000	1.786413000	0.645079000
C	-2.153110000	3.741485000	-1.172158000
H	-0.272457000	2.694003000	-1.244220000
C	-3.824827000	2.809341000	0.294786000
H	-3.263823000	1.025699000	1.349276000
C	-3.431981000	3.786141000	-0.620371000
H	-1.842038000	4.496226000	-1.885789000
H	-4.815199000	2.842532000	0.734739000
H	-4.117746000	4.578898000	-0.897607000
H	-2.154153000	-2.456518000	1.967055000
H	-4.136993000	-3.745785000	1.259974000
N	-5.442145000	-3.477517000	-1.054067000
H	-5.595277000	-3.533116000	-2.050296000
H	-5.585339000	-4.366655000	-0.597977000
C	0.700515000	1.138476000	0.929782000
H	0.678465000	2.207623000	1.167975000

Structure S4b

C	1.434807000	1.464549000	1.019355000
C	0.745808000	0.531910000	0.309414000
C	1.722917000	-0.341533000	-0.363529000
N	2.983598000	0.179753000	0.017624000
C	4.251963000	-0.430499000	-0.100139000
C	4.521565000	-1.274780000	-1.186971000
C	5.252301000	-0.172537000	0.846472000
C	5.782118000	-1.850052000	-1.308750000
H	3.742437000	-1.483286000	-1.904772000
C	6.512073000	-0.748014000	0.699128000
H	5.038692000	0.450640000	1.705094000
C	6.785766000	-1.589331000	-0.376115000
H	5.979855000	-2.506929000	-2.148409000
H	7.277030000	-0.543272000	1.439856000
H	7.765834000	-2.038819000	-0.484638000
O	1.561471000	-1.291352000	-1.104718000
C	0.973133000	2.602803000	1.867385000
H	1.579410000	3.497221000	1.691545000
H	-0.066261000	2.847449000	1.656932000
H	1.062237000	2.347521000	2.927767000
C	-0.721817000	0.327804000	0.225783000
N	-1.149831000	-0.842132000	0.521518000
C	-2.474884000	-1.277360000	0.345029000
C	-3.075911000	-2.010665000	1.377432000
C	-3.182903000	-1.130742000	-0.856835000
C	-4.361587000	-2.517991000	1.243321000
C	-4.455846000	-1.666861000	-1.004668000
H	-2.721074000	-0.615619000	-1.690149000
C	-5.074608000	-2.357561000	0.046573000
H	-4.977642000	-1.550404000	-1.949654000
C	-1.547466000	1.512256000	-0.187622000
C	-1.142848000	2.314369000	-1.262931000
C	-2.708367000	1.863477000	0.515615000
C	-1.892152000	3.426088000	-1.642008000
H	-0.243448000	2.057076000	-1.811118000
C	-3.444456000	2.986517000	0.149952000
H	-3.032375000	1.254363000	1.350557000
C	-3.043221000	3.767807000	-0.934018000
H	-1.575189000	4.026426000	-2.487640000
H	-4.335457000	3.249658000	0.708973000
H	-3.622739000	4.637497000	-1.222707000
H	-2.517975000	-2.173885000	2.292351000
H	-4.812099000	-3.062661000	2.067354000
N	-6.385501000	-2.837069000	-0.079801000
H	-6.689268000	-3.009323000	-1.027376000
H	-6.621613000	-3.605741000	0.531224000
H	3.400409000	2.009392000	0.771508000
N	2.800186000	1.208817000	0.953490000

Structure S5a

C	-0.926494000	1.982933000	-1.133926000
C	-1.162011000	0.613703000	0.760675000
N	-2.224316000	0.538427000	-0.125826000
N	-2.040099000	1.359084000	-1.246812000
C	-3.395810000	-0.260561000	-0.052741000
C	-3.656919000	-1.054144000	1.072897000
C	-4.301981000	-0.246983000	-1.121265000
C	-4.818027000	-1.822194000	1.113859000
H	-2.959848000	-1.067605000	1.896229000
C	-5.455613000	-1.021721000	-1.059272000
H	-4.095369000	0.366826000	-1.985611000
C	-5.723318000	-1.813940000	0.055549000
H	-5.010846000	-2.432800000	1.988926000
H	-6.148501000	-1.003120000	-1.893146000
H	-6.623612000	-2.415820000	0.098008000
O	-1.022079000	0.015305000	1.807279000
C	-0.451299000	2.938277000	-2.175539000
H	-0.306655000	3.938115000	-1.752943000
H	0.510199000	2.617580000	-2.588920000
H	-1.179187000	2.998797000	-2.984790000
C	1.212955000	1.095604000	-0.044736000
N	1.291355000	-0.134033000	-0.359303000
C	2.510168000	-0.814469000	-0.578079000
C	3.289706000	-0.618805000	-1.718925000
C	2.850874000	-1.830293000	0.344175000
C	4.419071000	-1.400962000	-1.956202000
C	3.993132000	-2.602106000	0.096763000
C	4.768359000	-2.391367000	-1.039879000
H	4.268422000	-3.376306000	0.806252000
H	2.984851000	0.133592000	-2.438372000
H	5.009898000	-1.243552000	-2.850662000
C	2.371987000	2.044216000	0.198311000
H	2.044498000	3.073285000	0.015198000
H	3.170806000	1.824141000	-0.511252000
C	2.949679000	1.934547000	1.631348000
H	3.839255000	2.571880000	1.670820000
C	1.985565000	2.332133000	2.753407000
H	1.615722000	3.355034000	2.620755000
H	1.125630000	1.659950000	2.812719000
H	2.493137000	2.293283000	3.720711000
H	3.294168000	0.908949000	1.790305000
H	5.642496000	-3.009203000	-1.211616000
N	2.092177000	-1.986357000	1.500299000
H	1.152875000	-1.608489000	1.460382000
H	2.138902000	-2.895272000	1.936177000
C	-0.202003000	1.641049000	0.147316000
H	-0.172154000	2.518956000	0.801669000

Structure S5b

C	-1.028028000	2.062215000	-0.702289000
C	-0.231363000	1.006830000	-0.384758000
C	-1.090051000	-0.184234000	-0.256360000
N	-2.391402000	0.282906000	-0.536330000
C	-3.623554000	-0.357849000	-0.269031000
C	-3.722919000	-1.750036000	-0.394674000
C	-4.748900000	0.393785000	0.091202000
C	-4.943448000	-2.371784000	-0.152669000
H	-2.849171000	-2.325455000	-0.661891000
C	-5.965624000	-0.245825000	0.315840000
H	-4.666790000	1.465677000	0.216209000
C	-6.070841000	-1.629305000	0.197659000
H	-5.011734000	-3.449728000	-0.246301000
H	-6.830518000	0.344875000	0.596142000
H	-7.018182000	-2.123646000	0.377825000
O	-0.820855000	-1.326201000	0.075617000
C	-0.706182000	3.480665000	-1.041935000
H	-0.949623000	4.148427000	-0.209745000
H	0.350684000	3.593244000	-1.278535000
H	-1.283465000	3.815464000	-1.909545000
C	1.229887000	0.943605000	-0.169326000
N	1.812083000	-0.049878000	-0.721656000
C	3.171282000	-0.377368000	-0.575796000
C	4.218945000	0.385296000	-1.094893000
C	3.439547000	-1.642663000	0.004649000
C	5.535695000	-0.071111000	-1.034369000
C	4.768456000	-2.080906000	0.069210000
C	5.804462000	-1.305552000	-0.445424000
H	4.984036000	-3.043132000	0.523612000
H	3.990129000	1.326670000	-1.582780000
H	6.337170000	0.526748000	-1.452116000
C	1.883150000	1.949432000	0.769846000
H	1.274186000	2.856028000	0.822640000
H	2.859708000	2.234172000	0.374945000
C	2.077055000	1.391777000	2.200185000
H	2.651443000	2.134211000	2.764978000
C	0.777844000	1.075997000	2.946587000
H	0.128473000	1.955722000	3.009460000
H	0.215876000	0.273380000	2.463077000
H	0.994072000	0.751560000	3.968038000
H	2.699468000	0.494419000	2.147004000
H	6.823107000	-1.673375000	-0.392011000
N	2.390526000	-2.368822000	0.549613000
H	1.458672000	-2.106319000	0.241515000
H	2.533798000	-3.363172000	0.637222000
H	-2.912866000	1.972230000	-1.514004000
N	-2.363224000	1.673479000	-0.712001000

Structure S6a

C	-1.262764000	2.013584000	-1.112151000
C	-1.448467000	0.650059000	0.790710000
N	-2.461782000	0.466148000	-0.137375000
N	-2.311554000	1.292673000	-1.259081000
C	-3.563132000	-0.428002000	-0.093264000
C	-3.751386000	-1.286479000	0.999019000
C	-4.473452000	-0.446440000	-1.158479000
C	-4.845166000	-2.148589000	1.010973000
H	-3.051538000	-1.274925000	1.820015000
C	-5.558488000	-1.316246000	-1.126134000
H	-4.321815000	0.215613000	-1.998239000
C	-5.754152000	-2.172298000	-0.043985000
H	-4.981602000	-2.808539000	1.860498000
H	-6.254846000	-1.321327000	-1.957362000
H	-6.601474000	-2.847802000	-0.024245000
O	-1.305420000	0.089101000	1.856802000
C	-0.833392000	2.998793000	-2.145389000
H	-0.789674000	4.010175000	-1.727638000
H	0.166396000	2.757077000	-2.519592000
H	-1.532969000	2.991444000	-2.981395000
C	0.908132000	1.323582000	0.046253000
N	1.099907000	0.165619000	-0.441183000
C	2.384450000	-0.365787000	-0.704483000
C	2.862543000	-1.428788000	0.080429000
C	3.106465000	0.094265000	-1.814645000
C	4.109349000	-1.998904000	-0.251962000
C	4.327486000	-0.491661000	-2.130020000
C	4.834784000	-1.525529000	-1.354447000
C	1.974746000	2.304272000	0.498945000
H	1.606620000	3.326480000	0.356289000
H	2.855416000	2.181917000	-0.133336000
C	2.407312000	2.119745000	1.974187000
H	3.251661000	2.795292000	2.147459000
C	1.318317000	2.386427000	3.018146000
H	0.905494000	3.396067000	2.913960000
H	0.496612000	1.669453000	2.948568000
H	1.733499000	2.309597000	4.026672000
H	2.797448000	1.105194000	2.097338000
H	5.791376000	-1.975760000	-1.599001000
C	2.078458000	-1.961312000	1.253624000
H	1.113333000	-1.466947000	1.348834000
H	1.881319000	-3.035745000	1.145798000
H	2.622734000	-1.827284000	2.195962000
N	4.650631000	-3.005146000	0.553930000
H	3.991368000	-3.566373000	1.071840000
H	5.369675000	-3.564105000	0.118977000
H	2.695101000	0.884886000	-2.430542000
H	4.886952000	-0.141059000	-2.990352000
C	-0.555760000	1.747675000	0.195812000
H	-0.623450000	2.628612000	0.842203000

Structure S6b

C	-1.355168000	2.094209000	-0.499223000
C	-0.547362000	1.033073000	-0.243546000
C	-1.383194000	-0.178609000	-0.226506000
N	-2.687754000	0.285397000	-0.510896000
C	-3.911578000	-0.396136000	-0.322244000
C	-3.975361000	-1.780102000	-0.535818000
C	-5.065371000	0.307329000	0.046285000
C	-5.188199000	-2.441097000	-0.372072000
H	-3.080535000	-2.319331000	-0.808705000
C	-6.273341000	-0.370637000	0.191921000
H	-5.012354000	1.370986000	0.238736000
C	-6.343116000	-1.746057000	-0.013880000
H	-5.228371000	-3.512448000	-0.533603000
H	-7.159975000	0.183407000	0.479282000
H	-7.283965000	-2.270558000	0.104638000
O	-1.098298000	-1.333972000	0.030975000
C	-1.046034000	3.538982000	-0.715955000
H	-1.389482000	4.144506000	0.128405000
H	0.025041000	3.691366000	-0.839834000
H	-1.545080000	3.916127000	-1.614503000
C	0.918124000	1.010280000	-0.016101000
N	1.583509000	0.208159000	-0.751722000
C	2.983196000	0.051302000	-0.672220000
C	3.504326000	-1.175956000	-0.222981000
C	3.830768000	1.058199000	-1.158042000
C	4.903091000	-1.352333000	-0.226049000
C	5.206180000	0.853770000	-1.168019000
C	5.746424000	-0.336150000	-0.697909000
C	1.484476000	1.864064000	1.112271000
H	0.823727000	2.717431000	1.290561000
H	2.458071000	2.255741000	0.812172000
C	1.660475000	1.084890000	2.437158000
H	2.153094000	1.758728000	3.146735000
C	0.362539000	0.554788000	3.053487000
H	-0.357750000	1.362277000	3.221854000
H	-0.113629000	-0.196547000	2.419063000
H	0.563417000	0.086266000	4.020961000
H	2.356026000	0.258031000	2.267851000
H	6.820982000	-0.488360000	-0.697123000
C	2.599124000	-2.284350000	0.253995000
H	1.549360000	-2.033957000	0.105923000
H	2.791373000	-3.216598000	-0.293127000
H	2.751529000	-2.500317000	1.318480000
N	5.457859000	-2.527743000	0.292895000
H	4.865368000	-3.344182000	0.272732000
H	6.397420000	-2.730399000	-0.015244000
H	3.403230000	1.974988000	-1.546053000
H	5.863353000	1.628659000	-1.547751000
H	-3.208824000	2.032816000	-1.377448000
N	-2.684419000	1.686531000	-0.577291000

Structure S7a

C	1.138372000	1.381569000	1.677563000
C	1.488777000	0.828510000	-0.577970000
N	2.430385000	0.293959000	0.289278000
N	2.184816000	0.644728000	1.623010000
C	3.550127000	-0.526847000	-0.004048000
C	3.805342000	-0.957024000	-1.313750000
C	4.410183000	-0.908887000	1.034311000
C	4.915044000	-1.759863000	-1.566100000
H	3.144593000	-0.664692000	-2.114956000
C	5.512131000	-1.712192000	0.759831000
H	4.206063000	-0.577617000	2.041866000
C	5.774517000	-2.143028000	-0.539296000
H	5.103125000	-2.087412000	-2.582559000
H	6.169005000	-2.001589000	1.572667000
H	6.634755000	-2.768643000	-0.747325000
O	1.443299000	0.717768000	-1.784395000
C	0.617400000	1.914880000	2.968589000
H	0.584642000	3.009471000	2.955358000
H	-0.401844000	1.560008000	3.150982000
H	1.255020000	1.590034000	3.790990000
C	-0.929902000	1.194056000	0.191146000
N	-1.136421000	-0.058901000	0.255631000
C	-2.431665000	-0.628233000	0.189020000
C	-3.302478000	-0.607900000	1.283421000
C	-2.817235000	-1.338928000	-0.952874000
C	-4.534366000	-1.253446000	1.228141000
C	-4.053097000	-1.971803000	-1.013760000
H	-2.136523000	-1.393826000	-1.794595000
C	-4.934163000	-1.942010000	0.076233000
H	-4.335709000	-2.503432000	-1.917048000
H	-3.006060000	-0.098750000	2.193952000
H	-5.192367000	-1.222224000	2.091033000
N	-6.200957000	-2.538968000	-0.005136000
H	-6.276802000	-3.289258000	-0.676957000
H	-6.610507000	-2.795467000	0.881667000
C	-1.976067000	2.277212000	-0.000101000
H	-1.652706000	3.184167000	0.523299000
H	-2.909206000	1.949762000	0.460936000
C	-2.264247000	2.620458000	-1.482545000
H	-3.115232000	3.309900000	-1.491189000
C	-1.096543000	3.242616000	-2.254533000
H	-0.732167000	4.150871000	-1.761495000
H	-0.259569000	2.548940000	-2.363766000
H	-1.417550000	3.527572000	-3.260156000
H	-2.600782000	1.711650000	-1.989923000
C	0.532777000	1.631438000	0.316813000
H	0.629228000	2.689202000	0.052353000

Structure S7b

C	-1.199358000	1.912227000	-0.633792000
C	-0.469143000	0.829286000	-0.264853000
C	-1.382468000	-0.322463000	-0.170175000
N	-2.646854000	0.197195000	-0.536332000
C	-3.916495000	-0.386340000	-0.325723000
C	-4.064939000	-1.777515000	-0.416165000
C	-5.031610000	0.418263000	-0.058501000
C	-5.321937000	-2.343727000	-0.231771000
H	-3.200404000	-2.394351000	-0.611210000
C	-6.284416000	-0.166848000	0.109488000
H	-4.914634000	1.489702000	0.039009000
C	-6.438216000	-1.548287000	0.026096000
H	-5.427305000	-3.420814000	-0.297549000
H	-7.140259000	0.465506000	0.317798000
H	-7.413862000	-2.000068000	0.161713000
O	-1.182416000	-1.465278000	0.194484000
C	-0.789597000	3.310367000	-0.960973000
H	-1.132968000	4.009889000	-0.192687000
H	0.293718000	3.386964000	-1.041618000
H	-1.220673000	3.632534000	-1.914537000
C	0.988024000	0.744653000	0.003071000
N	1.632137000	-0.123537000	-0.675109000
C	3.021664000	-0.336085000	-0.534690000
C	3.968802000	0.574996000	-1.016644000
C	3.480880000	-1.555027000	-0.019547000
C	5.330701000	0.292203000	-0.955681000
C	4.840718000	-1.829701000	0.058511000
H	2.755852000	-2.286015000	0.319194000
C	5.791145000	-0.911599000	-0.409517000
H	5.170584000	-2.774509000	0.479668000
H	3.635831000	1.504486000	-1.465434000
H	6.043972000	1.016044000	-1.338146000
N	7.166113000	-1.171026000	-0.285675000
H	7.409240000	-2.150341000	-0.238205000
H	7.751468000	-0.680848000	-0.947028000
C	1.565194000	1.631311000	1.100859000
H	0.937409000	2.519476000	1.218273000
H	2.561587000	1.968627000	0.809116000
C	1.675925000	0.914212000	2.467498000
H	2.171191000	1.606211000	3.157673000
C	0.343175000	0.458205000	3.068543000
H	-0.355204000	1.295382000	3.171862000
H	-0.137001000	-0.310955000	2.459006000
H	0.499845000	0.035933000	4.065073000
H	2.345788000	0.056540000	2.359298000
H	-3.026259000	1.891180000	-1.565688000
N	-2.550950000	1.584922000	-0.719694000

Structure S8a

C	4.411463000	-0.669525000	1.986266000
C	5.489343000	-0.347466000	-0.077033000
N	5.607791000	-1.628538000	0.427331000
N	4.957848000	-1.781630000	1.662659000
C	6.261894000	-2.754536000	-0.140933000
C	6.965719000	-2.647841000	-1.348378000
C	6.198963000	-3.986442000	0.523503000
C	7.595315000	-3.773453000	-1.874263000
H	7.012747000	-1.701267000	-1.863770000
C	6.834669000	-5.097817000	-0.020110000
H	5.653989000	-4.061325000	1.452885000
C	7.536856000	-5.001475000	-1.219950000
H	8.136638000	-3.681190000	-2.809229000
H	6.776970000	-6.046100000	0.502558000
H	8.029723000	-5.871049000	-1.638932000
O	5.941546000	0.090041000	-1.116386000
C	3.645640000	-0.507546000	3.253381000
H	4.076283000	0.288400000	3.869939000
H	2.610099000	-0.233878000	3.034464000
H	3.656637000	-1.440132000	3.817691000
C	3.420689000	1.084746000	0.388717000
N	2.405470000	0.325613000	0.246175000
C	1.215315000	0.689304000	-0.414453000
C	-0.000085000	0.405929000	0.216881000
C	1.212154000	1.178354000	-1.729009000
C	-1.215471000	0.689355000	-0.414489000
C	-0.000034000	1.402067000	-2.374813000
C	-1.212250000	1.178400000	-1.729045000
H	-0.000129000	-0.047722000	1.200586000
H	-2.149755000	1.347392000	-2.243666000
H	-0.000012000	1.755387000	-3.400019000
H	2.149686000	1.347317000	-2.243591000
N	-2.405615000	0.325713000	0.246165000
C	-3.420911000	1.084776000	0.388553000
C	-4.411571000	-0.669354000	1.986309000
C	-5.489340000	-0.347734000	-0.077109000
N	-4.957760000	-1.781587000	1.662809000
N	-5.607630000	-1.628761000	0.427408000
C	-6.261493000	-2.754930000	-0.140785000
C	-6.198572000	-3.986720000	0.523867000
C	-6.965081000	-2.648506000	-1.348392000
C	-6.834050000	-5.098249000	-0.019697000
H	-5.653780000	-4.061399000	1.453371000
C	-7.594451000	-3.774269000	-1.874225000
H	-7.012109000	-1.702016000	-1.863942000
C	-7.535998000	-5.002178000	-1.219700000
H	-6.776361000	-6.046441000	0.503138000
H	-8.135589000	-3.682213000	-2.809318000
H	-8.028685000	-5.871873000	-1.638642000
O	-5.941554000	0.089581000	-1.116538000

C	-3.645842000	-0.507090000	3.253444000
H	-2.610300000	-0.233411000	3.034538000
H	-4.076559000	0.288955000	3.869819000
H	-3.656819000	-1.439566000	3.817935000
C	3.520267000	2.545057000	0.080833000
C	4.647586000	3.061185000	-0.573261000
C	2.508753000	3.423266000	0.493390000
C	4.744113000	4.426922000	-0.831111000
H	5.428637000	2.386851000	-0.907064000
C	2.621599000	4.789236000	0.254803000
H	1.637743000	3.034972000	1.006743000
C	3.735917000	5.294254000	-0.414541000
H	5.610923000	4.812704000	-1.355698000
H	1.838707000	5.460388000	0.589518000
H	3.818781000	6.358050000	-0.606775000
C	-3.520609000	2.545040000	0.080498000
C	-2.509208000	3.423391000	0.493025000
C	-4.647939000	3.060986000	-0.573724000
C	-2.622172000	4.789324000	0.254283000
H	-1.638196000	3.035234000	1.006479000
C	-4.744583000	4.426685000	-0.831730000
H	-5.428896000	2.386534000	-0.907510000
C	-3.736497000	5.294159000	-0.415190000
H	-1.839368000	5.460591000	0.588971000
H	-5.611395000	4.812328000	-1.356417000
H	-3.819451000	6.357926000	-0.607546000
C	4.669040000	0.421481000	0.978337000
H	5.321983000	1.186190000	1.412596000
C	-4.669221000	0.421469000	0.978210000
H	-5.322294000	1.186169000	1.412287000

Structure S8b

C	5.343891000	0.173174000	1.517207000
C	4.636604000	0.062636000	0.361508000
C	5.261884000	-0.990244000	-0.457715000
N	6.356469000	-1.438392000	0.321130000
C	7.120718000	-2.615287000	0.156524000
C	7.362162000	-3.114927000	-1.131232000
C	7.663362000	-3.268438000	1.270881000
C	8.137279000	-4.259595000	-1.285182000
H	6.927505000	-2.617738000	-1.985433000
C	8.448028000	-4.405558000	1.094246000
H	7.450699000	-2.907065000	2.268605000
C	8.689653000	-4.908792000	-0.181358000
H	8.312823000	-4.643601000	-2.283805000
H	8.859769000	-4.904566000	1.964403000
H	9.296338000	-5.796827000	-0.314194000
O	4.976222000	-1.411812000	-1.560967000
C	5.182732000	1.078585000	2.692930000
H	6.149835000	1.468656000	3.026191000

H	4.540601000	1.922472000	2.447746000
H	4.738665000	0.535868000	3.532968000
C	3.408504000	0.789098000	-0.046682000
N	2.404526000	0.069136000	-0.374647000
C	1.216169000	0.554903000	-0.946836000
C	0.000012000	0.157658000	-0.380113000
C	1.212543000	1.286069000	-2.144933000
C	-1.216140000	0.554924000	-0.946831000
C	0.000022000	1.628035000	-2.736064000
C	-1.212503000	1.286074000	-2.144940000
H	0.000006000	-0.477611000	0.497362000
H	-2.150781000	1.541236000	-2.621091000
H	0.000026000	2.166391000	-3.677779000
H	2.150827000	1.541227000	-2.621076000
N	-2.404491000	0.069170000	-0.374614000
C	-3.408483000	0.789107000	-0.046626000
C	-5.343788000	0.173029000	1.517301000
C	-5.261818000	-0.990276000	-0.457692000
C	-4.636547000	0.062584000	0.361565000
N	-6.356429000	-1.438421000	0.321118000
C	-7.120720000	-2.615279000	0.156434000
C	-7.663384000	-3.268486000	1.270748000
C	-7.362181000	-3.114824000	-1.131355000
C	-8.448088000	-4.405569000	1.094036000
H	-7.450710000	-2.907187000	2.268496000
C	-8.137336000	-4.259457000	-1.285382000
H	-6.927507000	-2.617593000	-1.985523000
C	-8.689730000	-4.908709000	-0.181601000
H	-8.859845000	-4.904622000	1.964160000
H	-8.312892000	-4.643390000	-2.284030000
H	-9.296445000	-5.796716000	-0.314497000
O	-4.976214000	-1.411737000	-1.560999000
C	-5.182606000	1.078369000	2.693076000
H	-4.540201000	1.922087000	2.448031000
H	-6.149667000	1.468704000	3.026153000
H	-4.738853000	0.535513000	3.533190000
C	3.472683000	2.288896000	-0.037924000
C	4.593354000	2.946734000	-0.562234000
C	2.439735000	3.055256000	0.518061000
C	4.669078000	4.337462000	-0.553628000
H	5.401045000	2.364526000	-0.991162000
C	2.527083000	4.444011000	0.547911000
H	1.570210000	2.558408000	0.930024000
C	3.638071000	5.090217000	0.006325000
H	5.533961000	4.832538000	-0.980980000
H	1.725188000	5.024152000	0.990707000
H	3.700108000	6.172529000	0.022037000
C	-3.472701000	2.288905000	-0.037733000
C	-2.439805000	3.055251000	0.518372000
C	-4.593375000	2.946757000	-0.562019000
C	-2.527197000	4.444001000	0.548341000

H	-1.570281000	2.558398000	0.930328000
C	-4.669143000	4.337482000	-0.553294000
H	-5.401030000	2.364564000	-0.991032000
C	-3.638182000	5.090221000	0.006765000
H	-1.725337000	5.024128000	0.991218000
H	-5.534025000	4.832568000	-0.980636000
H	-3.700253000	6.172529000	0.022569000
H	-7.252129000	-0.501923000	1.874575000
H	7.252214000	-0.501809000	1.874505000
N	6.329736000	-0.805075000	1.572671000
N	-6.329664000	-0.805194000	1.572704000

Structure S9a

C	-4.236661000	0.078823000	2.009236000
C	-5.320718000	-0.215031000	-0.053297000
N	-5.452340000	1.057504000	0.478610000
N	-4.790201000	1.193133000	1.706138000
C	-6.114351000	2.189075000	-0.067401000
C	-6.826840000	2.096150000	-1.270943000
C	-6.051835000	3.412170000	0.613174000
C	-7.465189000	3.226059000	-1.776536000
H	-6.872910000	1.156566000	-1.799117000
C	-6.696467000	4.528156000	0.089819000
H	-5.499814000	3.477404000	1.539195000
C	-7.407247000	4.445337000	-1.105964000
H	-8.012758000	3.144204000	-2.708854000
H	-6.638643000	5.469505000	0.624917000
H	-7.906860000	5.318428000	-1.509361000
O	-5.778993000	-0.638603000	-1.093467000
C	-3.450607000	-0.096945000	3.263113000
H	-3.881399000	-0.886310000	3.888267000
H	-2.420701000	-0.386493000	3.032547000
H	-3.437008000	0.834453000	3.829398000
C	-3.218537000	-1.607899000	0.376805000
N	-2.418722000	-0.783251000	-0.168760000
C	-1.213839000	-1.165675000	-0.792513000
C	0.000282000	-0.860562000	-0.168800000
C	-1.212528000	-1.725401000	-2.077181000
C	1.214273000	-1.165637000	-0.792733000
C	0.000062000	-1.991425000	-2.707271000
C	1.212749000	-1.725327000	-2.077427000
H	0.000339000	-0.368069000	0.796157000
C	-2.999567000	-3.099406000	0.538706000
H	-3.291679000	-3.376775000	1.559553000
H	-1.928792000	-3.293391000	0.443052000
C	-3.749980000	-4.019426000	-0.455977000
H	-3.355309000	-5.029666000	-0.302685000
C	-5.275833000	-4.051262000	-0.323776000
H	-5.582399000	-4.299741000	0.698441000
H	-5.731691000	-3.100140000	-0.605852000

H	-5.693311000	-4.818509000	-0.981446000
H	-3.474678000	-3.735945000	-1.475360000
H	2.152777000	-1.908864000	-2.582813000
H	-0.000030000	-2.407790000	-3.708684000
H	-2.152634000	-1.908998000	-2.582401000
N	2.419342000	-0.783222000	-0.169280000
C	3.218593000	-1.607766000	0.377241000
C	4.237036000	0.078916000	2.009331000
C	5.320981000	-0.215232000	-0.053218000
N	4.790417000	1.193232000	1.705964000
N	5.452434000	1.057448000	0.478391000
C	6.114417000	2.188947000	-0.067808000
C	6.051946000	3.412120000	0.612629000
C	6.826810000	2.095885000	-1.271398000
C	6.696538000	4.528047000	0.089098000
H	5.499994000	3.477459000	1.538684000
C	7.465120000	3.225738000	-1.777167000
H	6.872846000	1.156239000	-1.799461000
C	7.407230000	4.445092000	-1.106727000
H	6.638752000	5.469459000	0.624091000
H	8.012619000	3.143781000	-2.709516000
H	7.906813000	5.318136000	-1.510261000
O	5.779378000	-0.639012000	-1.093247000
C	3.451159000	-0.096739000	3.263339000
H	2.421210000	-0.386292000	3.032956000
H	3.882028000	-0.886066000	3.888485000
H	3.437643000	0.834700000	3.829558000
C	2.998945000	-3.099058000	0.540264000
H	1.928051000	-3.292641000	0.445123000
H	3.291344000	-3.375971000	1.561142000
C	3.748611000	-4.019911000	-0.454221000
H	3.353927000	-5.030000000	-0.299961000
H	3.472692000	-3.737119000	-1.473626000
C	5.274544000	-4.051783000	-0.322922000
H	5.581702000	-4.299732000	0.699246000
H	5.691575000	-4.819415000	-0.980425000
H	5.730320000	-3.100839000	-0.605745000
C	-4.486695000	-0.994170000	0.976968000
H	-5.119918000	-1.780188000	1.399515000
C	4.486975000	-0.994203000	0.977181000
H	5.120039000	-1.780319000	1.399754000

Structure S9b

C	4.702194000	-1.696327000	0.761640000
C	4.343665000	-0.490028000	0.254652000
C	5.478961000	0.030583000	-0.525004000
N	6.465217000	-0.978551000	-0.430742000
C	7.841765000	-0.876180000	-0.734529000
C	8.258319000	-0.070855000	-1.803673000
C	8.786938000	-1.597480000	0.005982000

C	9.612296000	0.007146000	-2.112481000
H	7.526866000	0.491695000	-2.364512000
C	10.136814000	-1.516759000	-0.327794000
H	8.470379000	-2.198031000	0.848819000
C	10.558392000	-0.714790000	-1.385209000
H	9.927205000	0.636580000	-2.937167000
H	10.860256000	-2.077853000	0.253152000
H	11.610011000	-0.651246000	-1.638990000
O	5.628117000	1.097820000	-1.089067000
C	3.927405000	-2.702778000	1.546724000
H	4.300770000	-2.771717000	2.573010000
H	2.871367000	-2.438228000	1.577169000
H	4.010813000	-3.698546000	1.099057000
C	3.054797000	0.231722000	0.404762000
N	2.434436000	0.507381000	-0.674254000
C	1.215961000	1.215070000	-0.706758000
C	0.000012000	0.533044000	-0.575116000
C	1.213697000	2.585165000	-0.999173000
C	-1.215944000	1.215062000	-0.706736000
C	-0.000002000	3.253441000	-1.136000000
C	-1.213694000	2.585157000	-0.999151000
H	0.000016000	-0.537644000	-0.405123000
C	2.605484000	0.616679000	1.807746000
H	2.980270000	-0.124077000	2.521157000
H	1.513584000	0.590170000	1.844451000
C	3.074836000	2.020621000	2.258967000
H	2.628659000	2.207819000	3.242206000
C	4.592610000	2.204757000	2.344891000
H	5.050516000	1.452713000	2.995986000
H	5.067259000	2.139454000	1.363279000
H	4.832271000	3.188005000	2.759159000
H	2.649376000	2.766027000	1.581783000
H	-2.156174000	3.099404000	-1.141752000
H	-0.000008000	4.313074000	-1.368298000
H	2.156171000	3.099418000	-1.141789000
N	-2.434410000	0.507360000	-0.674209000
C	-3.054797000	0.231787000	0.404815000
C	-4.702244000	-1.696177000	0.761927000
C	-5.478882000	0.030499000	-0.525111000
C	-4.343657000	-0.489976000	0.254735000
N	-6.465190000	-0.978579000	-0.430670000
C	-7.841718000	-0.876228000	-0.734541000
C	-8.786946000	-1.597371000	0.006053000
C	-8.258202000	-0.071086000	-1.803851000
6	-10.136803000	-1.516682000	-0.327806000
H	-8.470448000	-2.197773000	0.849019000
C	-9.612161000	0.006887000	-2.112742000
H	-7.526708000	0.491346000	-2.364755000
6	-10.558310000	-0.714896000	-1.385388000
1	-10.860287000	-2.077654000	0.253206000
H	-9.927014000	0.636178000	-2.937558000

1	-11.609915000	-0.651376000	-1.639235000
O	-5.628001000	1.097644000	-1.089355000
C	-3.927545000	-2.702479000	1.547294000
H	-2.871387000	-2.438339000	1.577161000
H	-4.300520000	-2.770652000	2.573774000
H	-4.011550000	-3.698481000	1.100266000
C	-2.605532000	0.616892000	1.807774000
H	-1.513632000	0.590437000	1.844505000
H	-2.980297000	-0.123816000	2.521245000
C	-3.074961000	2.020853000	2.258855000
H	-2.628845000	2.208150000	3.242103000
H	-2.649492000	2.766218000	1.581633000
C	-4.592746000	2.204942000	2.344681000
H	-5.050663000	1.452938000	2.995815000
H	-4.832466000	3.188217000	2.758850000
H	-5.067339000	2.139537000	1.363048000
H	6.196031000	-2.916759000	0.063372000
N	6.026286000	-1.986980000	0.440285000
H	-6.196104000	-2.916697000	0.063860000
N	-6.026324000	-1.986844000	0.440570000
